# A Novel Evidence-Based Framework for Picture Fuzzy Sets: Theory and Applications of Belief and Plausibility

**DOI:** 10.3390/e28080918

**Published:** 2026-08-16

**Authors:** Rashid Hussain, Zahid Hussain, Mehboob Ali, Małgorzata Przybyła-Kasperek

**Affiliations:** 1 Department of Mathematical Sciences, Karakoram International University, Gilgit 15100, Pakistan; rashid.ali@kiu.edu.pk (R.H.); zahid.hussain@kiu.edu.pk (Z.H.); 2 Department of Statistics, Government College Gilgit, Gilgit 15100, Pakistan; 3 Institute of Computer Science, University of Silesia in Katowice, Bedzinska 39, 41-200 Sosnowiec, Poland

**Keywords:** picture fuzzy sets, belief and plausibility, distance and similarity measure, fault detection, clustering, MCDM, BP-SMART

## Abstract

Picture Fuzzy Sets (PiFSs) have appeared as an effective tool to tackle ambiguity in decision-making and offer greater flexibility than traditional extensions of Fuzzy Sets (FSs). Under the framework of evidence theory (ET), the concepts of belief and plausibility significantly boost the representative capacity of PiFSs, which enables the management of uncertain and ambiguous data. We constructed both distance and similarity measures specifically for Belief and Plausible Picture Fuzzy Sets (BP-PiFSs). The constructed measures detect the differences and connections between BP-PiFSs and addressed the key shortcomings in current methodologies. They are mathematically validated and applied to real-world scenarios, such as fault detection in complex systems and antenna design optimization, where managing uncertainty is critical. A modified decision-making method, Belief and Plausible SMART (BP-SMART), extends the classical SMART approach to more effectively handle multi-criteria decision-making (MCDM) in uncertain environments. Numerical evaluations across pattern recognition, clustering, fault detection, and MCDM demonstrates the effectiveness and robustness of the suggested framework, contributing significantly to both the theoretical and practical development of fuzzy set theory.

## 1. Introduction

The rapid growth of the social sector has resulted in a substantial increase in the volume of information generated across various professions. Consequently, many real-world datasets contain vague, imprecise, or ambiguous information that cannot always be effectively modeled using conventional approaches. Although probability theory provides a powerful framework for handling randomness and uncertainty, it is often inadequate for representing vagueness arising from imprecise information, thereby motivating the development of fuzzy set theory. Also, belief and plausibility measures [1,2] are some new approaches for coping with ambiguities. Since ET assigns probabilities to the power set of a given event [3], it is well suited for handling uncertainty and ambiguity. It is applied in a wide range of contexts, like clustering, classification [4,5], decision-making [6], knowledge reasoning [7], and risk assessment [8,9]. The aim of Shafer’s evidence theory [2] is to make the probability theory simple. In contrast, two-valued logic is insufficient for solving complex problems. To address this limitation, Zadeh [10] introduced fuzzy set (FS) as an extension of crisp sets (CS). The membership degree (MD) together with the non-membership degree (NMD) provides the foundational description of a FS. Fuzzy set theory (FST) provides a new way to communicate ambiguous and unreliable data while removing the shortcomings of traditional data management strategies. Research on fuzzy set theory has so far yielded important results and been widely used in many different fields. Some types of situations were encountered in handling different kind of uncertainties which were not in capacity of FSs to deal with them. As an illustration, FSs have MD and NMD with the condition: non-membership = 1−membership. It is sometimes not possible in daily life settings. Therefore, [11] generalized the notion of FSs to Intuitionistic Fuzzy Sets (IFSs), by comprising the NMD and hesitancy. Mathematically, IFSs are presented as follows: the MD μ, the NMD ν and degree of hesitancy π such that μ+ν+π=1 or 0≤μ+ν≤1, where π=1−μ−ν. So, the characterization of IFS is important due to their wide applicability in everyday life [12,13].

Numerous researchers extended classical FSs from various angles, such as IFSs [14], Pythagorean Fuzzy Sets (PyFSs) [15,16], and Hesitant Fuzzy Sets (HFSs) [17]. Among these, IFSs have received considerable attention. The inclusion of both MD and NMD in IFSs is the most distinctive feature, enabling them to represent vague, ambiguous, and uncertain information more effectively [11]. IFSs are effective methods for working with ambiguous data, and as a result, they are widely used in many different fields [18,19]. The sum of MD and NMD is less than or equal to one in IFS conceptual terms. These conditions, however, can be challenging to meet in some circumstances, and there are some circumstances that IFSs are unable to manage. For instance, voters can choose to vote for, vote against, abstain, or decline. Therefore, [20,21] introduced the picture fuzzy set (PiFS) to address such issues. It is fairer to handle issues like voting problems with Picture Fuzzy Sets, which are made up of membership, non-membership, and neutrality degrees that all range inside [0, 1] and add up to no more than 1. A significant advantage of PiFSs is the addition of a “neutrality” degree, which enhances the framework for complex decision-making in domains where a “maybe” or “neutral” posture is pertinent, such as medical diagnosis, hiring decisions, and social choice. For example, consider a survey in which 50% of respondents support a proposal, 20% oppose it, 15% remain neutral, and 15% do not provide any opinion. Using an IFS, the neutral and non-response groups would be combined into a single hesitation degree, making it impossible to distinguish between respondents who are genuinely neutral and those who are uncertain or unwilling to answer. As a result, important information about respondents’ attitudes is lost. PiFSs address this limitation by introducing a separate neutral membership degree. Thus, a PiFS consists of four components: positive membership, neutral membership, negative membership, and refusal (or abstention) degree. This allows positive, neutral, negative, and unknown opinions to be represented separately. For instance, when evaluating a project, an IFS representation such as membership = 0.6, non-membership = 0.2, and hesitation = 0.2 only indicates that 20% of the assessment is uncertain, without specifying the reason. In contrast, a PFS representation such as positive membership = 0.6, neutral membership = 0.1, negative membership = 0.2, and refusal degree = 0.1 clearly shows that 60% support the project, 10% are neutral, 20% oppose it, and 10% either lack information or refuse to express an opinion. This richer representation provides a more accurate reflection of real human judgments.

### 1.1. Literature Review

Many generalizations of ET were established such as [3,22,23] after FSs [24]. A generalized intuitionistic fuzzy belief and plausible function is presented by [25]. Refs. [26,27] presented a linkage between IFSs and ET. Since ref. [26,27] did not address the generalized form of belief and plausibility measure on IFS. Ref. [28] interpreted the belief and plausibility measures on IFSs and defining distances and similarities based on Hausdorff distance. Ref. [29] presented some similarities for belief function. A detailed analysis on belief and plausibility with applications is presented by [30]. Distance and similarities using belief and plausible measures is given by [31].

In FST, which has attracted considerable attention in recent decades, distance and similarity measures are widely used to quantify the differences between two FSs. Various distances and similarities between FSs, IFSs, and PiFSs are present in different contexts. For example, ref. [32] gave several distance measures for IFSs. Ref. [33] suggested similarity measures for IFSs using Jaccard index. Similarity measures along with applications are given by [34,35,36]. Ref. [6] extracted IFS distance with applications. Ref. [37] presented IFS based similarity with application in software quality evaluation. Distance measures on IFSs using cross-information dissimilarity is proposed by [38]. Ref. [39] suggested IFS based non-linear strict distance and similarity. Ref. [40] used similarity measure in face recognition and assessment of software quality. IFS based similarity measures with applications to pattern recognition is presented by [41]. Many researchers put forward distance and similarities of PiFSs. Zhao et al. [42] proposed distance and dissimilarity measures for PiFSs. Ganie [43] coined distance measure of PiFSs with applications. Zhu and Liu [44] proposed picture fuzzy distance measure with applications. Zhu et al. [45,46] coined distance measures for PiFSs with applications. Luo and Zhang [47] gave divergence-based distance of PiFSs. Hussain et al. [48] suggested decision algorithm for PiFSs. Wei and Gao [49] coined dice similarity for PiFSs with applications. Liu et al. [50] used interval-valued PiFSs and proposed similarity measures. Luo and Li, and Luo and Zhang [51,52] suggested some similarity measures on PiFSs. Verma and Rohtagi [53] coined similarity measures with application to pattern identifications. Rani et al. [54] suggested similarity measures with application to multi attribute decision making. Refs. [55,56] briefly studied PiFSs. Although, these approaches have had varied degrees of success, they nevertheless have a number of drawbacks:

- Several existing distance and similarity measures for PiFSs do not meet all axiomatic conditions.
- Some distance and similarity measures for PiFSs gave inconsistent or illogical results on distinct PiFSs.

However, to the best of our knowledge, there is no comprehensive framework that combines belief and plausibility measures with PiFSs.

### 1.2. Our Contribution

The main contributions of this paper can be summarized as:

- This paper constructs distance and similarity measures for BP-PiFSs by interpreting belief and plausibility functions into essential parameters (MD, NMD, degree of hesitancy, and degree of neutrality) of PiFSs.
- This paper also provides an effective framework to model and analyze difficult ambiguities in various contexts. The proposed measures are thoroughly authenticated for crucial mathematical properties, ensuring practical soundness in applications.

The application of our suggested measures to real-world problems includes fault detection using pattern recognition techniques, clustering and antenna design utilizing BP-SMART algorithm involving complex MCDM process demonstrates their utility and effectiveness in addressing practical challenges. Furthermore, this research lays a foundation for further advancements in generalization of FSs by integrating belief and plausibility measures in the context of PiFSs, encouraging investigation of other useful extensions and applications of the suggested measures.

### 1.3. Organization

Section 2 is dedicated to review fundamental definitions of IFSs, PyFSs and PiFSs. Section 3 interprets and extends the notion of belief and plausibility to PiFSs. Also, a new BP-distance is constructed. Section 4 deals with the construction of BP-similarity measures. Section 5 consists of applications of suggested measures to pattern recognition, fault detection, clustering, and MCDM. Also, we establish BP-SMART based on proposed BP-distance and BP-similarity measures to solve complicated decision-making scenarios. Section 6 includes the conclusion.

## 2. Basic Concepts

The fundamental concepts of IFSs, PyFSs, and PiFSs, respectively, are briefly discussed in this section.

**Definition** **1** ([14])**.** *Suppose* U *is a universe of discourse (UOD), and* A *is an IFS, then it will be shown as:*

$$A=\left\{u,\mu_{A}\left(u\right),\;v_{A}\left(u\right)\;:u\in U\;\right\}.$$

*In this definition,* $\mu_{A}\left(u\right):U\to [0,1]$ *is the MD,* $v_{A}\left(u\right):U\to [0,1]$ *is the NMD, obeying the condition* $0\;\leq \mu_{A}\left(u\right)+\;v_{A}\left(u\right)\leq 1$*. Here,* $\pi_{A}(u)=1-\left(\mu_{A}\left(u\right)+\;v_{A}\left(u\right)\right)$ *is known as degree of uncertainty.*

**Definition** **2** ([36])**.** *Suppose* A *is a PyFS in* U*, then it will be expressed as:*

$$A=\left\{u,\mu_{A}\left(u\right),\;v_{A}\left(u\right)\;:u\in U\right\}.$$

*In this definition, * $\mu_{A}\left(u\right):U\to [0,1]$ *is the MD, * $v_{A}\left(u\right):U\to [0,1]$ *is the NMD having the restriction* $0\;\leq \mu_{A}^{2}\left(u\right)+\;v_{A}^{2}\left(u\right)\leq 1$ *and* $\pi_{A}^{2}(u)=\sqrt{1-\left(\mu_{A}^{2}\left(u\right)+\;v_{A}^{2}\left(u\right)\right)}$ *is known as degree of uncertainty.*

**Definition** **3** ([53])**.** *Suppose* A *is a PiFS in* U*, it will be expressed as:*

$$A=\left\{u,\mu_{A}\left(u\right),\;v_{A}\left(u\right),\eta_{A}\left(u\right)\;:u\in U\;\right\}.$$

*Here, * $\mu_{A}\left(u\right):U\to [0,1]$ *is the MD, * $v_{A}\left(u\right):U\to [0,1]$ *is the NMD and* $\eta_{A}\left(u\right)\;$ *is the neutral degree (ND) of an element* u *to the PiFS* A *with restriction* $0\;\leq \mu_{A}\left(u\right)+\;v_{A}\left(u\right)+\eta_{A}\left(u\right)\leq 1$ *. For any* $\pi_{A}(u)=\sqrt{1-\left(\mu_{A}\left(u\right)+\;v_{A}\left(u\right)+\eta_{A}\left(u\right)\right)}\;:u\;\in \;U\;$ *is known as degree of uncertainty.*

**Remark** **1.** *The PiFS* A *becomes an IFS whenever* $\eta_{A}\left(u\right)=0\;\forall \;u\in U$.

**Definition** **4** ([47])**.** *Suppose* $A_{1}$ *and* $A_{2}$ *are any two PiFSs in* U*, some fundamental properties are:*

(i) $A_{1}\subseteq A_{2}\;iff$ $\mu_{A_{1}}\left(u\right)\leq \mu_{A_{2}}\left(u\right)$, $v_{A_{1}}\left(u\right)\geq v_{A_{2}}\left(u\right)$, and $\mu_{A_{1}}\left(u\right)+\eta_{A_{1}}\left(u\right)\leq \mu_{A_{2}}\left(u\right)+\eta_{A_{2}}\left(u\right)$;
(ii) $A_{1}\cup A_{2}=\left\{\begin{array}{l} max\left(\mu_{A_{1}}\left(u\right),\mu_{A_{2}}\left(u\right)\right),min\left(\nu_{A_{1}}\left(u\right),\nu_{A_{2}}\left(u\right)\right),\\ max\left(\mu_{A_{1}}\left(u\right)+\eta_{A_{1}}\left(u\right),\mu_{A_{2}}\left(u\right)+\eta_{A_{2}}\left(u\right)\right)-max\left(\mu_{A_{1}}\left(u\right),\mu_{A_{2}}\left(u\right)\right) \end{array}\right\}$;
(iii) $A_{1}\cap A_{2}=\left\{\begin{array}{l} min\left(\mu_{A_{1}}\left(u\right),\mu_{A_{2}}\left(u\right)\right),max\left(\nu_{A_{1}}\left(u\right),\nu_{A_{2}}\left(u\right)\right),\\ min\left(\mu_{A_{1}}\left(u\right)+\eta_{A_{1}}\left(u\right),\mu_{A_{2}}\left(u\right)+\eta_{A_{2}}\left(u\right)\right)-min\left(\mu_{A_{1}}\left(u\right),\mu_{A_{2}}\left(u\right)\right) \end{array}\right\}$;
(iv) $A_{1}=A_{2}\;iff\;\mu_{A_{1}}\left(u\right)=\mu_{A_{2}}\left(u\right)$, $v_{A_{1}}\left(u\right)=v_{A_{2}}\left(u\right)$ and $\eta_{A_{1}}\left(u\right)=\eta_{A_{2}}\left(u\right)$;
(v) ${\left(A_{1}\right)}^{c}=\left\{\;v_{A_{1}}\left(u\right),\;\mu_{A_{1}}\left(u\right),1-\mu_{A_{1}}\left(u\right)-v_{A_{1}}\left(u\right)-\eta_{A}\left(u\right)\right\}$.

**Figure 1:**
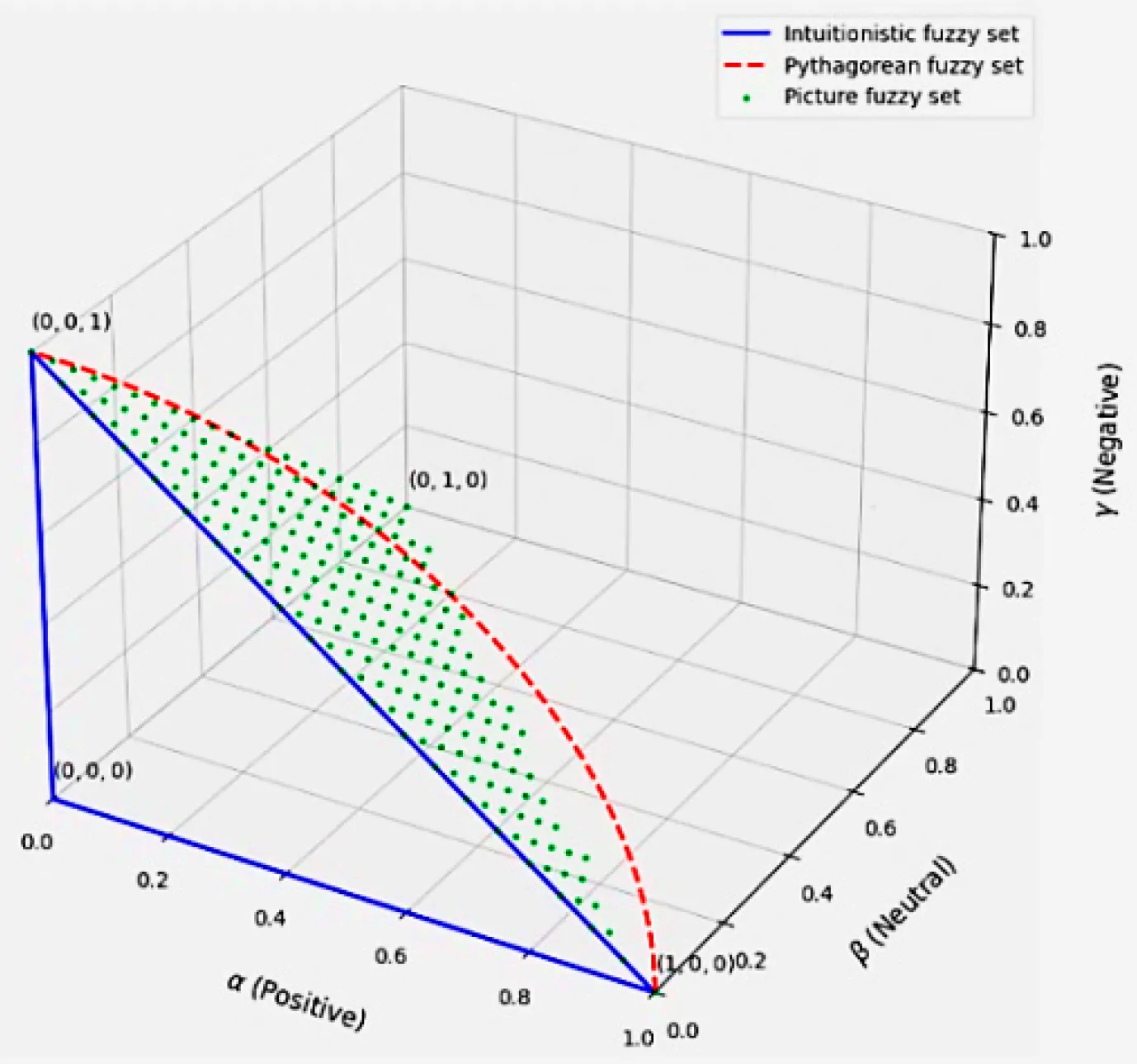
Visualization of IFSs, PyFSs, and PiFSs.

A three-dimensional visualization of IFSs, PFSs and PiFSs is demonstrated in Figure 1.

Next, a simple approach is presented to link belief and plausibility function to PiFSs.

## 3. Analysis of Belief and Plausibility Measures Within Picture Fuzzy Sets

According to the ET, belief is the whole amount of evidence that supports a statement. It is a lower bound that shows the lowest level of belief that the evidence supports. On the other hand, the upper bound, or plausibility, indicates how much the evidence does not contradict the argument. It measures how well the proposition is supported by the evidence.

These interpretations provide an organized approach to deal the uncertainty and ambiguity in reasoning and decision-making systems, bridging the gap between PiFSs and ET.

### 3.1. Relationship of ET with PiFSs

ET and PiFSs represent two different mathematical approaches to handle uncertainty and imprecision. PiFSs model uncertainty by assigning MD, NMD, and neutral degree to describe an element’s belongingness, non-belongingness, and neutrality, whereas ET addresses uncertainty using belief and plausibility functions. A closer analysis reveals that PiFSs and ET are fundamentally connected and share a strong relationship.

When interpreting ET in terms of PiFSs, the belief function $B_{A}\left(u\right)$ corresponds to the membership degree, which reflects the degree of support for the occurrence of an event. MD and belief are similar, i.e., $\mu_{A}\left(u\right)=B_{A}\left(u\right)$, which indicates the extent to which the element is believed to be part of the set.

In a similar manner, the plausibility function $P_{A}\left(u\right)$ indicates how strongly an element’s membership is not contradicted. It can be understood as the combination of $\mu_{A}\left(u\right)$ and $\pi_{A}\left(u\right)$, representing the possibility of belongingness of the element to the set under uncertainty. Mathematically, it is written as:


$$P_{A}\left(u\right)=\mu_{A}\left(u\right)+\pi_{A}\left(u\right)=1-\nu_{A}\left(u\right)-\eta_{A}\left(u\right).$$

**Definition** **5.** *Let* A *is a PiFS in* U*, then a belief function* $B_{A}\left(u\right)$ *is written as:*

(1) $$B_{A}\left(u\right)=\left\{\mu_{A}\left(u\right),\;\forall \;u\in \;U\right\}$$

**Definition** **6.** *Let* A *be any PiFS in* U*. A plausible function* $P_{F}\left(u\right)$ *is expressed as:*

(2) $$P_{A}\left(u\right)=\left\{1-v_{A}\left(u\right)-\eta_{A}\left(u\right)\;,\;\forall \;u\in U\right\}$$

Using Equations (1) and (2), we define BP-PiFS as:

**Definition** **7.** *Suppose* $BP_{A}$ *is a BP-PiFS in* U*, the mathematical form of* $BP_{A}$ *is constructed as:*

(3) $$BP_{A}=\left\{\langle u,\;B_{A}\left(u\right),\;P_{A}\left(u\right)\rangle :u\in U\right\}$$

*here* $0\leq B_{A}\left(u\right)\leq 1$*,* $0\leq P_{A}\left(u\right)\leq 1$ *and* $0\leq B_{A}\left(u\right)\leq P_{A}\left(u\right)\leq 1$ *with* $0\leq B_{A}\left(u\right)+P_{A}\left(u\right)\leq 2$.

For constructing new distance and similarity measures, Definition 7 is used.

### 3.2. Novel Distance Measure on BP-PiFSs

Distance measures play a crucial role in a wide range of disciplines by facilitating similarity assessment and supporting effective decision-making. In finance, health care, and business management, they help compare alternatives and improve systems such as recommendation models. In pattern recognition, distance measures facilitate data classification by comparing feature vectors. Similarly, in machine learning, they play a fundamental role in clustering algorithms, which are widely applied to tasks such as customer segmentation, document clustering, and social network analysis. In medical imaging, distance measures assist in monitoring disease progression and detecting abnormalities. Geographic information systems also rely on distance measures for mapping, route optimization, and proximity evaluation. Furthermore, distance measures are important in fraud detection, where they help identify anomalous patterns in transactional data. Various measures, including Euclidean and Manhattan distances, are designed to suit different types of applications and data structures.

A novel BP-distance measure is formulated to quantify the difference between two BP-PiFSs, $BP_{A_{1}}$ and $BP_{A_{2}}$, as defined below.

(4) $$\begin{array}{l} d\left(BP_{A_{1}},BP_{A_{2}}\right)=\frac{1}{n}\sum_{i=1}^{n}\left[2min\left\{\begin{array}{l} max\left(B_{A_{1}}\left(u_{i}\right),B_{A_{2}}\left(u_{i}\right)\right),\\ max\left(P_{A_{1}}\left(u_{i}\right),P_{A_{2}}\left(u_{i}\right)\right) \end{array}\right\}\right.\\ \;\;\;\;\;\;\;\;\;\;\;\;\;\;\;\;\;\;\;\;\;\;\;\;\;\;\;\;\;\;\;\;\;\;\;\;\;\;\;\;\;\;\;\;\;\;\;\;\;\;\;\;\;\;\left.-min\left\{\begin{array}{l} \left(B_{A_{1}}\left(u_{i}\right)+B_{A_{2}}\left(u_{i}\right)\right),\\ \left(P_{A_{1}}\left(u_{i}\right)+P_{A_{2}}\left(u_{i}\right)\right) \end{array}\right\}\right] \end{array}$$

Assigning of weights to available criteria can enhance the reliability of decision under set criteria. Let $0\leq w_{i}\leq 1$ with the condition that $\sum_{i=1}^{n}w_{i}=1$. Therefore, the weighted BP-distance is defined as:

(5) $$\begin{array}{l} d_{w}\left(BP_{A_{1}},BP_{A_{2}}\right)=\sum_{i=1}^{n}w_{i}\left[2min\left\{\begin{array}{l} max\left(B_{A_{1}}\left(u_{i}\right),B_{A_{2}}\left(u_{i}\right)\right),\\ max\left(P_{A_{1}}\left(u_{i}\right),P_{A_{2}}\left(u_{i}\right)\right) \end{array}\right\}\right.\\ \;\;\;\;\;\;\;\;\;\;\;\;\;\;\;\;\;\;\;\;\;\;\;\;\;\;\;\;\;\;\;\;\;\;\;\;\;\;\;\;\;\;\;\;\;\;\;\;\;\;\;\;\;\left.-min\left\{\begin{array}{l} \left(B_{A_{1}}\left(u_{i}\right)+B_{A_{2}}\left(u_{i}\right)\right),\\ \left(P_{A_{1}}\left(u_{i}\right)+P_{A_{2}}\left(u_{i}\right)\right) \end{array}\right\}\right] \end{array}$$

The above BP-distance measure is defined as:

**Definition** **8.** *Let* $BP_{A_{1}}$*, *$BP_{A_{2}}$ *and* $BP_{A_{3}}$ *are three BP-PiFSs in* U*. Then, a real-valued function* $d:\;PiFS\left(U\right)\times PiFS\left(U\right)\to \left[0,1\right]$* is known as distance measure if the following properties are satisfied:*

$\left(d_{1}\right):$$\;0\leq d\left(BP_{A_{1}},\;BP_{A_{2}}\right)\leq 1\;$ (Non-Negativity);
$\left(d_{2}\right):$ $d\left(BP_{A_{1}},\;BP_{A_{2}}\right)=0,\;if\;\mathrm{and}\;\mathrm{only}\;if\;BP_{A_{1}}=\;BP_{A_{2}}$ (separability);
$\left(d_{3}\right):\;$$d\left(BP_{A_{1}},\;BP_{A_{2}}\right)=d\;\left(BP_{A_{2}},BP_{A_{1}}\right)$ (symmetric);
$\left(d_{4}\right):$ $if\;BP_{A_{1}}\leq \;BP_{A_{2}}\leq BP_{A_{3}}\;$, then $d\left(BP_{A_{1}},\;BP_{A_{2}}\right)\leq d\left(BP_{A_{1}},\;BP_{A_{3}}\right)$ and $\;d\left(BP_{A_{2}},\;BP_{A_{3}}\right)\leq d\left(BP_{A_{1}},\;BP_{A_{3}}\right)$ (containment).
$\left(d_{5}\right):$ For any $BP_{A_{1}},BP_{A_{2}},BP_{A_{3}}$ on U, $d\left(BP_{A_{1}},\;BP_{A_{3}}\right)\leq d\left(BP_{A_{1}},\;BP_{A_{2}}\right)+d\left(BP_{A_{2}},\;BP_{A_{3}}\right)$ (triangular inequality).

The following theorem confirms the suitability of the proposed distance measure defined in Equation (4).

**Theorem** **1.** *Suppose* U *is an UOD, then the distance measure* $d\left(BP_{A_{1}},\;BP_{A_{2}}\right)$ *in Equation (4) between two BP-PiFSs* $BP_{A_{1}}$ *and* $BP_{A_{2}}$ *justifies the properties of Definition 8.*

**Property** **1** $\left(d_{1}\right)$**.** $\;0\leq d\left(BP_{A_{1}},\;BP_{A_{2}}\right)\leq 1\;$.

**Proof.**  Let us have two BP-PiFSs as $BP_{A_{1}}=\left\{u,\;B_{A_{1}}\left(u_{i}\right),\;P_{A_{1}}\left(u_{i}\right)\;\right\}$ and $BP_{A_{2}}=\left\{u,\;B_{A_{2}}\left(u_{i}\right),\;P_{A_{2}}\left(u_{i}\right)\;\right\}$ in $U=\left\{u_{1},u_{2},\ldots ,u_{n}\right\}$. The $d\left(BP_{A_{1}},\;BP_{A_{2}}\right)$ of Equation (4), is positive, that is $d\left(BP_{A_{1}},\;BP_{A_{2}}\right)\geq 0$,

$$\left[\begin{array}{l} 2min\left\{max\left(B_{A_{1}}\left(u_{i}\right),B_{A_{2}}\left(u_{i}\right)\right),max\left(P_{A_{1}}\left(u_{i}\right),P_{A_{2}}\left(u_{i}\right)\right)\right\}\\ -min\left\{\left(B_{A_{1}}\left(u_{i}\right)+B_{A_{2}}\left(u_{i}\right)\right),\left(P_{A_{1}}\left(u_{i}\right)+P_{A_{2}}\left(u_{i}\right)\right)\right\} \end{array}\right]\geq 0$$

which implies that

$$\frac{1}{n}\sum_{i=1}^{n}\left[\begin{array}{l} 2min\left\{max\left(B_{A_{1}}\left(u_{i}\right),B_{A_{2}}\left(u_{i}\right)\right),max\left(P_{A_{1}}\left(u_{i}\right),P_{A_{2}}\left(u_{i}\right)\right)\right\}\\ -min\left\{\left(B_{A_{1}}\left(u_{i}\right)+B_{A_{2}}\left(u_{i}\right)\right),\left(P_{A_{1}}\left(u_{i}\right)+P_{A_{2}}\left(u_{i}\right)\right)\right\} \end{array}\right]\geq 0$$

$$0\leq \frac{1}{n}\sum_{i=1}^{n}\left[\begin{array}{l} 2min\left\{max\left(B_{A_{1}}\left(u_{i}\right),B_{A_{2}}\left(u_{i}\right)\right),max\left(P_{A_{1}}\left(u_{i}\right),P_{A_{2}}\left(u_{i}\right)\right)\right\}\\ -min\left\{\left(B_{A_{1}}\left(u_{i}\right)+B_{A_{2}}\left(u_{i}\right)\right),\left(P_{A_{1}}\left(u_{i}\right)+P_{A_{2}}\left(u_{i}\right)\right)\right\} \end{array}\right]\leq 1$$

Hence $0\leq d\left(BP_{A_{1}},\;BP_{A_{2}}\right)\leq 1$. This satisfies the Property 1 $\left(d_{1}\right)$. □

**Property** **2** $\left(d_{2}\right)$**.** $d\left(BP_{A_{1}},\;BP_{A_{2}}\right)=0,\;if\;\mathrm{and}\;\mathrm{only}\;if\;BP_{A_{1}}=\;BP_{A_{2}}$.

**Proof.**  If $BP_{A_{1}}=BP_{A_{2}}$, then for each $u_{i}\in U$, $B_{A_{1}}(u_{i})=B_{A_{2}}(u_{i})$ and $P_{A_{1}}(u_{i})=P_{A_{2}}(u_{i})$.

$$\begin{array}{l} d\left(BP_{A_{1}},BP_{A_{2}}\right)=\frac{1}{n}\sum_{i=1}^{n}\left[2min\left\{max\left(B_{A_{1}}\left(u_{i}\right),B_{A_{1}}\left(u_{i}\right)\right),max\left(P_{A_{1}}\left(u_{i}\right),P_{A_{1}}\left(u_{i}\right)\right)\right\}\right.\\ \;\;\;\;\;\;\;\;\;\;\;\;\;\;\;\;\;\;\;\;\;\;\;\;\;\;\;\;\;\;\;\;\;\;\;\;\;\;\;\;\;\;\;\;\;\;\;\;\;\;\;\;\;\;\left.-min\left\{\left(B_{A_{1}}\left(u_{i}\right)+B_{A_{1}}\left(u_{i}\right)\right),\left(P_{A_{1}}\left(u_{i}\right)+P_{A_{1}}\left(u_{i}\right)\right)\right\}\right] \end{array}$$

$$d\left(BP_{A_{1}},\;BP_{A_{2}}\right)=\frac{1}{n}\sum_{i=1}^{n}\left[\begin{array}{l} 2min\left\{B_{A_{1}}\left(u_{i}\right),P_{A_{1}}\left(u_{i}\right)\right\}\\ -2min\left\{B_{A_{1}}\left(u_{i}\right),P_{A_{1}}\left(u_{i}\right)\right\} \end{array}\right]=\frac{1}{n}\sum_{i=1}^{n}\left[0\right]=0.$$

Thus, $d\left(BP_{A_{1}},\;BP_{A_{2}}\right)=0$. Conversely, if $d\left(BP_{A_{1}},\;BP_{A_{2}}\right)=0$, then $d\left(BP_{A_{1}},\;BP_{A_{2}}\right)=\frac{1}{n}\sum_{i=1}^{n}\left[0\right]$ implies that

$$d\left(BP_{A_{1}},\;BP_{A_{2}}\right)=\frac{1}{n}\sum_{i=1}^{n}\left[2min\left\{B_{A_{1}}\left(u_{i}\right),P_{A_{1}}\left(u_{i}\right)\right\}-2min\left\{B_{A_{1}}\left(u_{i}\right),P_{A_{1}}\left(u_{i}\right)\right\}\right],$$

$$\begin{array}{l} d\left(BP_{A_{1}},BP_{A_{2}}\right)=\frac{1}{n}\sum_{i=1}^{n}\left[2min\left\{max\left(B_{A_{1}}\left(u_{i}\right),B_{A_{1}}\left(u_{i}\right)\right),max\left(P_{A_{1}}\left(u_{i}\right),P_{A_{1}}\left(u_{i}\right)\right)\right\}\right.\\ \;\;\;\;\;\;\;\;\;\;\;\;\;\;\;\;\;\;\;\;\;\;\;\;\;\;\;\;\;\;\;\;\;\;\;\;\;\;\;\;\;\;\;\;\;\;\;\;\;\;\left.-min\left\{\left(B_{A_{1}}\left(u_{i}\right)+B_{A_{1}}\left(u_{i}\right)\right),\left(P_{A_{1}}\left(u_{i}\right)+P_{A_{1}}\left(u_{i}\right)\right)\right\}\right] \end{array}$$

which is possible only when $B_{A_{1}}\left(u_{i}\right)=B_{A_{2}}\left(u_{i}\right)$. Thus, $d\left(BP_{A_{1}},\;BP_{A_{2}}\right)=0$. This proves the Property 2 $\left(d_{2}\right)$ of Definition 8. □

**Property** **3** $\left(d_{3}\right)$**.** $d\left(BP_{A_{1}},\;BP_{A_{2}}\right)=\;d\left(BP_{A_{2}},BP_{A_{1}}\right)\;$.

**Proof.**  Since

$$\begin{array}{l} d\left(BP_{A_{1}},BP_{A_{2}}\right)=\frac{1}{n}\sum_{i=1}^{n}\left[2min\left\{max\left(B_{A_{1}}\left(u_{i}\right),B_{A_{2}}\left(u_{i}\right)\right),max\left(P_{A_{1}}\left(u_{i}\right),P_{A_{2}}\left(u_{i}\right)\right)\right\}\right.\\ \;\;\;\;\;\;\;\;\;\;\;\;\;\;\;\;\;\;\;\;\;\;\;\;\;\;\;\;\;\;\;\;\;\;\;\;\;\;\;\;\;\;\;\;\;\;\;\;\;\;\;\left.-min\left\{\left(B_{A_{1}}\left(u_{i}\right)+B_{A_{2}}\left(u_{i}\right)\right),\left(P_{A_{1}}\left(u_{i}\right)+P_{A_{2}}\left(u_{i}\right)\right)\right\}\right] \end{array},$$

$$\begin{array}{l} =\frac{1}{n}\sum_{i=1}^{n}\left[2min\left\{max\left(B_{A_{2}}\left(u_{i}\right),B_{A_{1}}\left(u_{i}\right)\right),max\left(P_{A_{2}}\left(u_{i}\right),P_{A_{1}}\left(u_{i}\right)\right)\right\}\right.\\ \;\;\;\;\;\;\;\;\;\;\;\;\;\;\;\;\;\;\;\;\;\;\;\left.-min\left\{\left(B_{A_{2}}\left(u_{i}\right)+B_{A_{1}}\left(u_{i}\right)\right),\left(P_{A_{2}}\left(u_{i}\right)+P_{A_{1}}\left(u_{i}\right)\right)\right\}\right] \end{array}\;,$$

$=\;d\left(BP_{A_{2}},BP_{A_{1}}\right)\;$ for each $u_{i}\in U.$ Thus, Property 3 $\left(d_{3}\right)$ is satisfied. □

**Property** **4** $\left(d_{4}\right)$**.** $if\;BP_{A_{1}}\leq \;BP_{A_{2}}\leq BP_{A_{3}}\;$*, then*

$$d\left(BP_{A_{1}},\;BP_{A_{2}}\right)\leq d\left(BP_{A_{1}},\;BP_{A_{3}}\right)$$

and

$$d\left(BP_{A_{2}},\;BP_{A_{3}}\right)\leq d\left(BP_{A_{1}},\;BP_{A_{3}}\right).$$

**Proof.**  Let $BP_{A_{1}}\subseteq BP_{A_{2}}\subseteq BP_{A_{3}}$, so $B_{A_{1}}(u_{i})\leq B_{A_{2}}(u_{i})\leq B_{A_{3}}(u_{i})$ and $P_{A_{1}}(u_{i})\geq P_{A_{2}}(u_{i})\geq P_{A_{3}}(u_{i})$$\forall \;\;u_{i}\in U$. From Equation (4), we have

$$\begin{array}{l} 2min\left\{max\left(B_{A_{1}}\left(u_{i}\right),B_{A_{3}}\left(u_{i}\right)\right),max\left(P_{A_{1}}\left(u_{i}\right),P_{A_{3}}\left(u_{i}\right)\right)\right\}\\ -min\left\{\left(B_{A_{1}}\left(u_{i}\right)+B_{A_{3}}\left(u_{i}\right)\right),\left(P_{A_{1}}\left(u_{i}\right)+P_{A_{3}}\left(u_{i}\right)\right)\right\}\geq \\ \;2min\left\{max\left(B_{A_{1}}\left(u_{i}\right),B_{A_{2}}\left(u_{i}\right)\right),max\left(P_{A_{1}}\left(u_{i}\right),P_{A_{2}}\left(u_{i}\right)\right)\right\}\\ -min\left\{\left(B_{A_{1}}\left(u_{i}\right)+B_{A_{2}}\left(u_{i}\right)\right),\left(P_{A_{1}}\left(u_{i}\right)+P_{A_{2}}\left(u_{i}\right)\right)\right\} \end{array}$$

which implies that

$$\begin{array}{l} \frac{1}{n}\sum_{i=1}^{n}\left[\begin{array}{l} 2min\left\{max\left(B_{A_{1}}\left(u_{i}\right),B_{A_{3}}\left(u_{i}\right)\right),max\left(P_{A_{1}}\left(u_{i}\right),P_{A_{3}}\left(u_{i}\right)\right)\right\}\\ -min\left\{\left(B_{A_{1}}\left(u_{i}\right)+B_{A_{3}}\left(u_{i}\right)\right),\left(P_{A_{1}}\left(u_{i}\right)+P_{A_{3}}\left(u_{i}\right)\right)\right\} \end{array}\right]\geq \\ \frac{1}{n}\sum_{i=1}^{n}\left[\begin{array}{l} 2min\left\{max\left(B_{A_{1}}\left(u_{i}\right),B_{A_{2}}\left(u_{i}\right)\right),max\left(P_{A_{1}}\left(u_{i}\right),P_{A_{2}}\left(u_{i}\right)\right)\right\}\\ -min\left\{\left(B_{A_{1}}\left(u_{i}\right)+B_{A_{2}}\left(u_{i}\right)\right),\left(P_{A_{1}}\left(u_{i}\right)+P_{A_{2}}\left(u_{i}\right)\right)\right\} \end{array}\right] \end{array}$$

So, $\;d\left(BP_{A_{1}},BP_{A_{3}}\right)\geq d\left(BP_{A_{1}},BP_{A_{2}}\right)$. Similarly,

$$\begin{array}{l} 2min\left\{max\left(B_{A_{1}}\left(u_{i}\right),B_{A_{3}}\left(u_{i}\right)\right),max\left(P_{A_{1}}\left(u_{i}\right),P_{A_{3}}\left(u_{i}\right)\right)\right\}\\ -min\left\{\left(B_{A_{1}}\left(u_{i}\right)+B_{A_{3}}\left(u_{i}\right)\right),\left(P_{A_{1}}\left(u_{i}\right)+P_{A_{3}}\left(u_{i}\right)\right)\right\}\geq \\ \;2min\left\{max\left(B_{A_{2}}\left(u_{i}\right),B_{A_{3}}\left(u_{i}\right)\right),max\left(P_{A_{2}}\left(u_{i}\right),P_{A_{3}}\left(u_{i}\right)\right)\right\}\\ -min\left\{\left(B_{A_{2}}\left(u_{i}\right)+B_{A_{3}}\left(u_{i}\right)\right),\left(P_{A_{2}}\left(u_{i}\right)+P_{A_{3}}\left(u_{i}\right)\right)\right\}\;, \end{array}$$

$$\begin{array}{l} \frac{1}{n}\sum_{i=1}^{n}\left[\begin{array}{l} 2min\left\{max\left(B_{A_{1}}\left(u_{i}\right),B_{A_{3}}\left(u_{i}\right)\right),max\left(P_{A_{1}}\left(u_{i}\right),P_{A_{3}}\left(u_{i}\right)\right)\right\}\\ -min\left\{\left(B_{A_{1}}\left(u_{i}\right)+B_{A_{3}}\left(u_{i}\right)\right),\left(P_{A_{1}}\left(u_{i}\right)+P_{A_{3}}\left(u_{i}\right)\right)\right\} \end{array}\right]\geq \\ \frac{1}{n}\sum_{i=1}^{n}\left[\begin{array}{l} 2min\left\{max\left(B_{A_{2}}\left(u_{i}\right),B_{A_{3}}\left(u_{i}\right)\right),max\left(P_{A_{2}}\left(u_{i}\right),P_{A_{3}}\left(u_{i}\right)\right)\right\}\\ -min\left\{\left(B_{A_{2}}\left(u_{i}\right)+B_{A_{3}}\left(u_{i}\right)\right),\left(P_{A_{2}}\left(u_{i}\right)+P_{A_{3}}\left(u_{i}\right)\right)\right\} \end{array}\right] \end{array}$$

So, $\;d\left(BP_{A_{1}},BP_{A_{3}}\right)\geq d\left(BP_{A_{2}},BP_{A_{3}}\right)$. □

**Property** **5** $\left(d_{5}\right)$**.** *For any* $BP_{A_{1}},BP_{A_{2}},BP_{A_{3}}$*,*

$$d\left(BP_{A_{1}},\;BP_{A_{3}}\right)\leq d\left(BP_{A_{1}},\;BP_{A_{2}}\right)+d\left(BP_{A_{2}},\;BP_{A_{3}}\right)$$

**Proof.**  Suppose that $\left\{max\left(B_{A_{1}}\left(u_{i}\right),B_{A_{3}}\left(u_{i}\right)\right),max\left(P_{A_{1}}\left(u_{i}\right),P_{A_{3}}\left(u_{i}\right)\right)\right\}\;\leq$

$$\left\{max\left(B_{A_{1}}\left(u_{i}\right),B_{A_{2}}\left(u_{i}\right)\right),max\left(P_{A_{1}}\left(u_{i}\right),P_{A_{2}}\left(u_{i}\right)\right)\right\}\;,$$

then

$$\begin{array}{l} 2min\left\{max\left(B_{A_{1}}\left(u_{i}\right),B_{A_{3}}\left(u_{i}\right)\right),max\left(P_{A_{1}}\left(u_{i}\right),P_{A_{3}}\left(u_{i}\right)\right)\right\}\\ \;\;\;\;\;\;\;\;\;\;\;-min\left\{\left(B_{A_{1}}\left(u_{i}\right)+B_{A_{3}}\left(u_{i}\right)\right),\left(P_{A_{1}}\left(u_{i}\right)+P_{A_{3}}\left(u_{i}\right)\right)\right\}\;\leq \end{array}$$

$$\begin{array}{l} 2min\left\{max\left(B_{A_{1}}\left(u_{i}\right),B_{A_{2}}\left(u_{i}\right)\right),max\left(P_{A_{1}}\left(u_{i}\right),P_{A_{2}}\left(u_{i}\right)\right)\right\}\\ \;\;\;\;\;\;\;\;\;\;\;-min\left\{\left(B_{A_{1}}\left(u_{i}\right)+B_{A_{2}}\left(u_{i}\right)\right),\left(P_{A_{1}}\left(u_{i}\right)+P_{A_{2}}\left(u_{i}\right)\right)\right\} \end{array},$$

So,

$$\frac{1}{n}\sum_{i=1}^{n}\left[\begin{array}{l} 2min\left\{max\left(B_{A_{1}}\left(u_{i}\right),B_{A_{3}}\left(u_{i}\right)\right),max\left(P_{A_{1}}\left(u_{i}\right),P_{A_{3}}\left(u_{i}\right)\right)\right\}\\ -min\left\{\left(B_{A_{1}}\left(u_{i}\right)+B_{A_{3}}\left(u_{i}\right)\right),\left(P_{A_{1}}\left(u_{i}\right)+P_{A_{3}}\left(u_{i}\right)\right)\right\} \end{array}\right]\geq$$

$$\frac{1}{n}\sum_{i=1}^{n}\left[\begin{array}{l} 2min\left\{max\left(B_{A_{1}}\left(u_{i}\right),B_{A_{2}}\left(u_{i}\right)\right),max\left(P_{A_{1}}\left(u_{i}\right),P_{A_{2}}\left(u_{i}\right)\right)\right\}\\ -min\left\{\left(B_{A_{1}}\left(u_{i}\right)+B_{A_{2}}\left(u_{i}\right)\right),\left(P_{A_{1}}\left(u_{i}\right)+P_{A_{2}}\left(u_{i}\right)\right)\right\} \end{array}\right]$$

Hence,

(6) $$d\left(BP_{A_{1}},\;BP_{A_{3}}\right)\leq d\left(BP_{A_{1}},\;BP_{A_{2}}\right)$$

Similarly, if

$$\left\{max\left(B_{A_{1}}\left(u_{i}\right),B_{A_{3}}\left(u_{i}\right)\right),max\left(P_{A_{1}}\left(u_{i}\right),P_{A_{3}}\left(u_{i}\right)\right)\right\}\;\leq$$

$$\left\{max\left(B_{A_{2}}\left(u_{i}\right),B_{A_{3}}\left(u_{i}\right)\right),max\left(P_{A_{2}}\left(u_{i}\right),P_{A_{3}}\left(u_{i}\right)\right)\right\}\;,$$

then

$$\begin{array}{l} 2min\left\{max\left(B_{A_{1}}\left(u_{i}\right),B_{A_{3}}\left(u_{i}\right)\right),max\left(P_{A_{1}}\left(u_{i}\right),P_{A_{3}}\left(u_{i}\right)\right)\right\}\\ \;\;\;\;\;\;\;\;\;\;\;-min\left\{\left(B_{A_{1}}\left(u_{i}\right)+B_{A_{3}}\left(u_{i}\right)\right),\left(P_{A_{1}}\left(u_{i}\right)+P_{A_{3}}\left(u_{i}\right)\right)\right\}\;\leq \end{array}$$

$$\begin{array}{l} 2min\left\{max\left(B_{A_{2}}\left(u_{i}\right),B_{A_{3}}\left(u_{i}\right)\right),max\left(P_{A_{2}}\left(u_{i}\right),P_{A_{3}}\left(u_{i}\right)\right)\right\}\\ \;\;\;\;\;\;\;\;\;\;\;-min\left\{\left(B_{A_{2}}\left(u_{i}\right)+B_{A_{3}}\left(u_{i}\right)\right),\left(P_{A_{2}}\left(u_{i}\right)+P_{A_{3}}\left(u_{i}\right)\right)\right\} \end{array},$$

So,

$$\frac{1}{n}\sum_{i=1}^{n}\left[\begin{array}{l} 2min\left\{max\left(B_{A_{1}}\left(u_{i}\right),B_{A_{3}}\left(u_{i}\right)\right),max\left(P_{A_{1}}\left(u_{i}\right),P_{A_{3}}\left(u_{i}\right)\right)\right\}\\ -min\left\{\left(B_{A_{1}}\left(u_{i}\right)+B_{A_{3}}\left(u_{i}\right)\right),\left(P_{A_{1}}\left(u_{i}\right)+P_{A_{3}}\left(u_{i}\right)\right)\right\} \end{array}\right]\geq$$

$$\frac{1}{n}\sum_{i=1}^{n}\left[\begin{array}{l} 2min\left\{max\left(B_{A_{2}}\left(u_{i}\right),B_{A_{3}}\left(u_{i}\right)\right),max\left(P_{A_{2}}\left(u_{i}\right),P_{A_{3}}\left(u_{i}\right)\right)\right\}\\ -min\left\{\left(B_{A_{2}}\left(u_{i}\right)+B_{A_{3}}\left(u_{i}\right)\right),\left(P_{A_{2}}\left(u_{i}\right)+P_{A_{3}}\left(u_{i}\right)\right)\right\} \end{array}\right]$$

Hence,

(7) $$d\left(BP_{A_{1}},\;BP_{A_{3}}\right)\leq d\left(BP_{A_{2}},\;BP_{A_{3}}\right)$$

From Equations (6) and (7), it proves that

$$d\left(BP_{A_{1}},\;BP_{A_{3}}\right)\leq d\left(BP_{A_{1}},\;BP_{A_{2}}\right)+d\left(BP_{A_{2}},\;BP_{A_{3}}\right)$$

This completes the proof. □

## 4. Novel Similarity Measures on BP-PiFSs

Similarity measures play a significant role in distinguishing and evaluating different objects or sets, and they are broadly applied in fields including pattern recognition [57], cluster analysis [33,35] and MCDM [35].

The proposed similarity measure of $BP_{F_{1}}$ and $BP_{F_{2}}$ will be


(8) $$S\left(BP_{A_{1}}\;,\;BP_{A_{2}}\right)=\frac{1}{n}\sum_{i=1}^{n}\left[\frac{\left\{B_{A_{1}}^{2}\left(u_{i}\right)\cdot B_{A_{2}}^{2}\left(u_{i}\right)+P_{A_{1}}^{2}\left(u_{i}\right)\cdot P_{A_{2}}^{2}\left(u_{i}\right)\right\}}{max\left(B_{A_{1}}^{4}\left(u_{i}\right),B_{A_{2}}^{4}\left(u_{i}\right)\right)+max\left(P_{A_{1}}^{4}\left(u_{i}\right),P_{A_{2}}^{4}\left(u_{i}\right)\right)}\right]$$

**Definition** **9.** *Let* $BP_{A_{1}}$*, * $BP_{A_{2}}$ *and* $BP_{A_{3}}$ *be any three BP-PiFSs* U*. Then, a real-valued function* $S:PiFS\left(U\right)\times PiFS\left(U\right)\to \left[0,1\right]$ *is known as similarity measure between two BP-PiFSs if it fulfills the below properties:*

$\left(s_{1}\right):$$\;0\leq S\left(BP_{A_{1}},\;\;BP_{A_{2}}\right)\leq 1$;
$\left(s_{2}\right):$ $S\left(BP_{A_{1}},\;BP_{A_{2}}\right)=0,\;iff\;BP_{A_{1}}=\;BP_{A_{2}}$;
$\left(s_{3}\right):\;$$S\left(BP_{A_{1}},\;BP_{A_{2}}\right)=S\;\left(BP_{A_{2}},BP_{A_{1}}\right)$;
$\left(s_{4}\right):$ $if\;BP_{A_{1}}\subseteq \;BP_{A_{2}}\subseteq BP_{A_{3}}\;$, then $S\left(BP_{A_{1}},\;\;BP_{A_{2}}\right)\leq S\left(BP_{A_{1}},\;\;BP_{A_{3}}\right)$ and $S\left(BP_{A_{2}},\;\;BP_{A_{3}}\right)\leq S\left(BP_{A_{1}},\;\;BP_{A_{3}}\right)$.

To test the appropriateness of proposed similarity measure $S\left(BP_{A_{1}},\;BP_{A_{2}}\right)$ of Equation (8), following theorem is provided.

**Theorem** **2.** *Suppose* U *is a universal discourse set. The proposed similarity measure* $S\left(BP_{A_{1}},\;BP_{A_{2}}\right)$ *in Equation (8) between two BP-PiFSs* $BP_{A_{1}}$ *and* $BP_{A_{2}}$ *fulfills the properties of Definition 9.*

**Property** **6** $\left(s_{1}\right)$**.** $\;0\leq S\left(BP_{A_{1}},\;BP_{A_{2}}\right)\leq 1\;\;$.

**Proof.**  Given that $BP_{A_{1}}=\left\{u,\;B_{A_{1}}\left(u_{i}\right),\;P_{A_{1}}\left(u_{i}\right)\;\right\}$ and $BP_{A_{2}}=\left\{u,\;B_{A_{2}}\left(u_{i}\right),\;P_{A_{2}}\left(u_{i}\right)\;\right\}$ are two BP-PiFSs in the finite universe $U=\left\{u_{1},u_{2},\ldots ,u_{n}\right\}$. Since $\left\{B_{A_{1}}^{2}\left(u_{i}\right)\cdot B_{A_{2}}^{2}\left(u_{i}\right)+P_{A_{1}}^{2}\left(u_{i}\right)\cdot P_{A_{2}}^{2}\left(u_{i}\right)\right\}\geq 0$, and $max\left(B_{A_{1}}^{4}\left(u_{i}\right),B_{A_{2}}^{4}\left(u_{i}\right)\right)+max\left(P_{A_{1}}^{4}\left(u_{i}\right),P_{A_{2}}^{4}\left(u_{i}\right)\right)\geq 0$, which implies that

$$\frac{\left\{B_{A_{1}}^{2}\left(u_{i}\right)\cdot B_{A_{2}}^{2}\left(u_{i}\right)+P_{A_{1}}^{2}\left(u_{i}\right)\cdot P_{A_{2}}^{2}\left(u_{i}\right)\right\}}{max\left(B_{A_{1}}^{4}\left(u_{i}\right),B_{A_{2}}^{4}\left(u_{i}\right)\right)+max\left(P_{A_{1}}^{4}\left(u_{i}\right),P_{A_{2}}^{4}\left(u_{i}\right)\right)}\geq 0$$

and

$$\frac{1}{n}\sum_{i=1}^{n}\left[\frac{\left\{B_{A_{1}}^{2}\left(u_{i}\right)\cdot B_{A_{2}}^{2}\left(u_{i}\right)+P_{A_{1}}^{2}\left(u_{i}\right)\cdot P_{A_{2}}^{2}\left(u_{i}\right)\right\}}{max\left(B_{A_{1}}^{4}\left(u_{i}\right),B_{A_{2}}^{4}\left(u_{i}\right)\right)+max\left(P_{A_{1}}^{4}\left(u_{i}\right),P_{A_{2}}^{4}\left(u_{i}\right)\right)}\right]\geq 0,$$

So,

$$0\leq \frac{1}{n}\sum_{i=1}^{n}\left[\frac{\left\{B_{A_{1}}^{2}\left(u_{i}\right)\cdot B_{A_{2}}^{2}\left(u_{i}\right)+P_{A_{1}}^{2}\left(u_{i}\right)\cdot P_{A_{2}}^{2}\left(u_{i}\right)\right\}}{max\left(B_{A_{1}}^{4}\left(u_{i}\right),B_{A_{2}}^{4}\left(u_{i}\right)\right)+max\left(P_{A_{1}}^{4}\left(u_{i}\right),P_{A_{2}}^{4}\left(u_{i}\right)\right)}\right]\leq 1$$

Hence, $0\leq S\left(BP_{A_{1}},\;BP_{A_{2}}\right)\leq 1$ which proves the Property 6 $\left(s_{1}\right)$. □

**Property** **7** $\left(s_{2}\right)$**.** $S\left(BP_{A_{1}},\;BP_{A_{2}}\right)=1,\;if\;\mathrm{and}\;\mathrm{only}\;if\;BP_{A_{1}}=\;BP_{A_{2}}$.

**Proof.**  If $BP_{A_{1}}=BP_{A_{2}}$, then for each $u_{i}\in U$, $B_{A_{1}}(u_{i})=B_{A_{2}}(u_{i})$ and $P_{A_{1}}(u_{i})=P_{A_{2}}(u_{i})$.

$$S\left(BP_{A_{1}}\;,\;BP_{A_{2}}\right)=\frac{1}{n}\sum_{i=1}^{n}\left[\frac{\left\{B_{A_{1}}^{2}\left(u_{i}\right)\cdot B_{A_{1}}^{2}\left(u_{i}\right)+P_{A_{1}}^{2}\left(u_{i}\right)\cdot P_{A_{1}}^{2}\left(u_{i}\right)\right\}}{max\left(B_{A_{1}}^{4}\left(u_{i}\right),B_{A_{1}}^{4}\left(u_{i}\right)\right)+max\left(P_{A_{1}}^{4}\left(u_{i}\right),P_{A_{1}}^{4}\left(u_{i}\right)\right)}\right],$$

$$S\left(BP_{A_{1}},\;BP_{A_{2}}\right)=\frac{1}{n}\sum_{i=1}^{n}\left[\frac{\left\{B_{A_{1}}^{4}\left(u_{i}\right)+P_{A_{1}}^{4}\left(u_{i}\right)\right\}}{B_{A_{1}}^{4}\left(u_{i}\right)+P_{A_{1}}^{4}\left(u_{i}\right)}\right]=\frac{1}{n}\sum_{i=1}^{n}\left[1\right]=1.$$

Thus, $S\left(BP_{A_{1}},\;BP_{A_{2}}\right)=1$. Conversely, if $S\left(BP_{A_{1}},\;BP_{A_{2}}\right)=1$, then

$$S\left(BP_{A_{1}},\;BP_{A_{2}}\right)=\frac{1}{n}\sum_{i=1}^{n}\left[1\right]$$

implies that $S\left(BP_{A_{1}},\;BP_{A_{2}}\right)=\frac{1}{n}\sum_{i=1}^{n}\left[\frac{\left\{B_{A_{1}}^{4}\left(u_{i}\right)+P_{A_{1}}^{4}\left(u_{i}\right)\right\}}{B_{A_{1}}^{4}\left(u_{i}\right)+P_{A_{1}}^{4}\left(u_{i}\right)}\right]$,

$$S\left(BP_{A_{1}}\;,\;BP_{A_{2}}\right)=\frac{1}{n}\sum_{i=1}^{n}\left[\frac{\left\{B_{A_{1}}^{2}\left(u_{i}\right)\cdot B_{A_{1}}^{2}\left(u_{i}\right)+P_{A_{1}}^{2}\left(u_{i}\right)\cdot P_{A_{1}}^{2}\left(u_{i}\right)\right\}}{max\left(B_{A_{1}}^{4}\left(u_{i}\right),B_{A_{1}}^{4}\left(u_{i}\right)\right)+max\left(P_{A_{1}}^{4}\left(u_{i}\right),P_{A_{1}}^{4}\left(u_{i}\right)\right)}\right]$$

which is only possible when $B_{A_{1}}\left(u_{i}\right)=B_{A_{2}}\left(u_{i}\right)$. Thus, $S\left(BP_{A_{1}},\;BP_{A_{2}}\right)=1$. This proves the Property 7 $\left(s_{2}\right)$ of Definition 9. □

**Property** **8** $\left(s_{3}\right)$**.** $S\left(BP_{A_{1}},\;BP_{A_{2}}\right)=\;S\left(BP_{A_{2}},BP_{A_{1}}\right)\;$.

**Proof.**  Since

$$S\left(BP_{A_{1}}\;,\;BP_{A_{2}}\right)=\frac{1}{n}\sum_{i=1}^{n}\left[\frac{\left\{B_{A_{1}}^{2}\left(u_{i}\right)\cdot B_{A_{2}}^{2}\left(u_{i}\right)+P_{A_{1}}^{2}\left(u_{i}\right)\cdot P_{A_{2}}^{2}\left(u_{i}\right)\right\}}{max\left(B_{A_{1}}^{4}\left(u_{i}\right),B_{A_{2}}^{4}\left(u_{i}\right)\right)+max\left(P_{A_{1}}^{4}\left(u_{i}\right),P_{A_{2}}^{4}\left(u_{i}\right)\right)}\right],$$

$$=\frac{1}{n}\sum_{i=1}^{n}\left[\frac{\left\{B_{A_{2}}^{2}\left(u_{i}\right)\cdot B_{A_{1}}^{2}\left(u_{i}\right)+P_{A_{2}}^{2}\left(u_{i}\right)\cdot P_{A_{1}}^{2}\left(u_{i}\right)\right\}}{max\left(B_{A_{2}}^{4}\left(u_{i}\right),B_{A_{1}}^{4}\left(u_{i}\right)\right)+max\left(P_{A_{2}}^{4}\left(u_{i}\right),P_{A_{1}}^{4}\left(u_{i}\right)\right)}\right]$$

$=\;S\left(BP_{A_{2}},BP_{A_{1}}\right)\;$ for each $u_{i}\in U.$ Hence, Property 8 $\left(s_{3}\right)$ of Definition 9 is satisfied. □

**Property** **9** $\left(s_{4}\right)$**.** $if\;BP_{A_{1}}\subseteq \;BP_{A_{2}}\subseteq BP_{A_{3}}\;$*, then*

$$S\left(BP_{A_{1}},\;BP_{A_{3}}\right)\leq S\left(BP_{A_{1}},\;BP_{A_{2}}\right)$$

and

$$\;S\left(BP_{A_{1}},\;BP_{A_{3}}\right)\leq S\left(BP_{A_{2}},\;BP_{A_{3}}\right).$$

**Proof.**  Let $BP_{A_{1}}\subseteq BP_{A_{2}}\subseteq BP_{A_{3}}$, so $B_{A_{1}}(u_{i})\leq B_{A_{2}}(u_{i})\leq B_{A_{3}}(u_{i})$ and $P_{A_{1}}(u_{i})\geq P_{A_{2}}(u_{i})\geq P_{A_{3}}(u_{i})$$\forall \;u_{i}\in U$. From Equation (8), we have

$$\frac{\left\{B_{A_{1}}^{2}\left(u_{i}\right)\cdot B_{A_{2}}^{2}\left(u_{i}\right)+P_{A_{1}}^{2}\left(u_{i}\right)\cdot P_{A_{2}}^{2}\left(u_{i}\right)\right\}}{max\left(B_{A_{1}}^{4}\left(u_{i}\right),B_{A_{2}}^{4}\left(u_{i}\right)\right)+max\left(P_{A_{1}}^{4}\left(u_{i}\right),P_{A_{2}}^{4}\left(u_{i}\right)\right)}\geq$$

$$\frac{\left\{B_{A_{1}}^{2}\left(u_{i}\right)\cdot B_{A_{3}}^{2}\left(u_{i}\right)+P_{A_{1}}^{2}\left(u_{i}\right)\cdot P_{A_{3}}^{2}\left(u_{i}\right)\right\}}{max\left(B_{A_{1}}^{4}\left(u_{i}\right),B_{A_{3}}^{4}\left(u_{i}\right)\right)+max\left(P_{A_{1}}^{4}\left(u_{i}\right),P_{A_{3}}^{4}\left(u_{i}\right)\right)}$$

which implies that

$$\frac{1}{n}\sum_{i=1}^{n}\left[\frac{\left\{B_{A_{1}}^{2}\left(u_{i}\right)\cdot B_{A_{2}}^{2}\left(u_{i}\right)+P_{A_{1}}^{2}\left(u_{i}\right)\cdot P_{A_{2}}^{2}\left(u_{i}\right)\right\}}{max\left(B_{A_{1}}^{4}\left(u_{i}\right),B_{A_{2}}^{4}\left(u_{i}\right)\right)+max\left(P_{A_{1}}^{4}\left(u_{i}\right),P_{A_{2}}^{4}\left(u_{i}\right)\right)}\right]\geq$$

$$\frac{1}{n}\sum_{i=1}^{n}\left[\frac{\left\{B_{A_{1}}^{2}\left(u_{i}\right)\cdot B_{A_{3}}^{2}\left(u_{i}\right)+P_{A_{1}}^{2}\left(u_{i}\right)\cdot P_{A_{3}}^{2}\left(u_{i}\right)\right\}}{max\left(B_{A_{1}}^{4}\left(u_{i}\right),B_{A_{3}}^{4}\left(u_{i}\right)\right)+max\left(P_{A_{1}}^{4}\left(u_{i}\right),P_{A_{3}}^{4}\left(u_{i}\right)\right)}\right],$$

So,

$$S\left(BP_{A_{1}},BP_{A_{2}}\right)\geq S\left(BP_{A_{1}},BP_{A_{3}}\right).$$

Similarly,

$$\frac{\left\{B_{A_{2}}^{2}\left(u_{i}\right)\cdot B_{A_{3}}^{2}\left(u_{i}\right)+P_{A_{2}}^{2}\left(u_{i}\right)\cdot P_{A_{3}}^{2}\left(u_{i}\right)\right\}}{max\left(B_{A_{2}}^{4}\left(u_{i}\right),B_{A_{3}}^{4}\left(u_{i}\right)\right)+max\left(P_{A_{2}}^{4}\left(u_{i}\right),P_{A_{3}}^{4}\left(u_{i}\right)\right)}\geq$$

$$\frac{\left\{B_{A_{1}}^{2}\left(u_{i}\right)\cdot B_{A_{3}}^{2}\left(u_{i}\right)+P_{A_{1}}^{2}\left(u_{i}\right)\cdot P_{A_{3}}^{2}\left(u_{i}\right)\right\}}{max\left(B_{A_{1}}^{4}\left(u_{i}\right),B_{A_{3}}^{4}\left(u_{i}\right)\right)+max\left(P_{A_{1}}^{4}\left(u_{i}\right),P_{A_{3}}^{4}\left(u_{i}\right)\right)}$$

which implies that

$$\frac{1}{n}\sum_{i=1}^{n}\left[\frac{\left\{B_{A_{2}}^{2}\left(u_{i}\right)\cdot B_{A_{3}}^{2}\left(u_{i}\right)+P_{A_{2}}^{2}\left(u_{i}\right)\cdot P_{A_{3}}^{2}\left(u_{i}\right)\right\}}{max\left(B_{A_{2}}^{4}\left(u_{i}\right),B_{A_{3}}^{4}\left(u_{i}\right)\right)+max\left(P_{A_{2}}^{4}\left(u_{i}\right),P_{A_{3}}^{4}\left(u_{i}\right)\right)}\right]\geq$$

$$\frac{1}{n}\sum_{i=1}^{n}\left[\frac{\left\{B_{A_{1}}^{2}\left(u_{i}\right)\cdot B_{A_{3}}^{2}\left(u_{i}\right)+P_{A_{1}}^{2}\left(u_{i}\right)\cdot P_{A_{3}}^{2}\left(u_{i}\right)\right\}}{max\left(B_{A_{1}}^{4}\left(u_{i}\right),B_{A_{3}}^{4}\left(u_{i}\right)\right)+max\left(P_{A_{1}}^{4}\left(u_{i}\right),P_{A_{3}}^{4}\left(u_{i}\right)\right)}\right],$$

Hence, $\;S\left(BP_{A_{2}},BP_{A_{3}}\right)\geq S\left(BP_{A_{1}},BP_{A_{3}}\right)$. Thus, the Property 9 $\left(s_{4}\right)$ of Definition 9 is satisfied, which completes the proofs. □

To demonstrate the effectiveness and reliability of the proposed distance and similarity measures in Equations (4) and (8), several numerical examples are provided. These examples illustrate their practical usefulness and confirm that the results accurately reflect similarity relationships.

## 5. Applications of the Proposed Distance and Similarity Measures

Here, we suggest some applications of our proposed measures of Equations (4) and (8) in fault detection and clustering to assess the practical utility of the suggested measures.

### 5.1. Application to Fault Detection

Fault detection is a critical component of modern systems, playing a vital role in various industries, technologies, and everyday applications. It involves identifying deviations from expected behavior in machinery, electronic systems, software, or biological systems, often before catastrophic failure occurs. Fault detection enables systems to identify when machinery operates outside its expected parameters, when software is behaving abnormally, or when sensors are providing incorrect readings. This ability to monitor and analyze data for irregularities allows for early intervention, preventing damage, improving safety, and minimizing downtime. Fault detection is essential as it allows systems to learn from historical data, identify patterns associated with faults, and make informed decisions in real time. Its growing importance is evident in fields such as industrial automation, aerospace, healthcare monitoring, and smart grids. For example, in manufacturing, algorithms can detect equipment failures by analyzing vibration or temperature data, while in health care, abnormal patterns in vital signs can indicate a medical issue. Fault detection has become an increasingly important and dynamic area of research over the past several decades, as it supports the development of intelligent, self-monitoring, and adaptive systems. By detecting faults early, organizations can prevent equipment damage, reduce downtime, ensure safety, and save millions in maintenance costs.

Now, using the proposed measures of Equations (4) and (8), we provide a fault detection case below.

**Example** **1.** *Detecting faults in rotating machinery is a critical task in mechanical engineering, especially in industries like manufacturing, aerospace, and automotive, where equipment reliability is very important. Pattern recognition techniques are commonly employed to classify machinery conditions based on vibration signal analysis. These signals are collected using sensors and analyzed to extract key features such as amplitude, frequency components, spectral entropy, harmonic distortion, and kurtosis values. Such features help differentiate between normal and faulty operating conditions. Consider a classification problem where motors are monitored in real-time, and their vibration data is used to detect and classify potential faults. The system identifies five classes of operational conditions:*

*A_1_: normal operation, A_2_: unbalance, A_3_: misalignment, A_4_: bearing defect, and A_5_: gear fault. Each class represents a distinct pattern in the vibration signal associated with specific mechanical issues. Using pattern recognition algorithms, the system can accurately categorize the machinery’s state, enable predictive maintenance and minimize downtime.*

*The five classes of operational conditions are represented by PiFSs* $A_{1}$, $A_{2}$, $A_{3}$, $A_{4}$*, and* $A_{5}$ *in the UOD* U *as:*

$$A_{1}=\left\{\langle u_{1},0.2,0.1,0.3\rangle ,\langle u_{2},0.1,0.3,0.1\rangle ,\langle u_{3},0.4,0.3,0.2\rangle \right\};$$

$$A_{2}=\left\{\langle u_{1},0.4,0.1,0.3\rangle ,\langle u_{2},0.3,0.5,0.1\rangle ,\langle u_{3},0.2,0.4,0.2\rangle \right\};$$

$$A_{3}=\left\{\langle u_{1},0.3,0.2,0.1\rangle ,\langle u_{2},0.6,0.1,0.3\rangle ,\langle u_{3},0.5,0.1,0.3\rangle \right\};$$

$$A_{4}=\left\{\langle u_{1},0.4,0.3,0.1\rangle ,\langle u_{2},0.5,0.3,0.1\rangle ,\langle u_{3},0.6,0.1,0.2\rangle \right\}$$

and

$$A_{5}=\left\{\langle u_{1},0.3,0.1,0.4\rangle ,\langle u_{2},0.5,0.2,0.2\rangle ,\langle u_{3},0.6,0.1,0.3\rangle \right\}.$$

*An unknown vibration signal taken as sample is*

$$S=\left\{\langle u_{1},0.4,0.1,0.1\rangle ,\langle u_{2},0.3,0.4,0.2\rangle ,\langle u_{3},0.6,0.1,0.2\rangle \right\}.$$

*This process aims to classify the unknown signal through similarity evaluation with predefined classes in order to detect the most probable mechanical fault or confirm normal system behavior. The corresponding BP-PiFSs of* $A_{1}$, $A_{2}$, $A_{3}$, $A_{4}$, $A_{5}$, *and* S *are* $BP_{A_{1}}$, $BP_{A_{2}}$, $BP_{A_{3}}$, $BP_{A_{4}}$, $BP_{A_{5}}$, *and* $BP_{S}$, *respectively, and are expressed as:*

$$BP_{A_{1}}=\left\{\langle u_{1},0.2,0.6\rangle ,\langle u_{2},0.1,0.6\rangle ,\langle u_{3},0.4,0.5\rangle \right\};$$

$$BP_{A_{2}}=\left\{\langle u_{1},0.4,0.6\rangle ,\langle u_{2},0.3,0.4\rangle ,\langle u_{3},0.2,0.4\rangle \right\};$$

$$BP_{A_{3}}=\left\{\langle u_{1},0.3,0.7\rangle ,\langle z_{2},0.6,0.6\rangle ,\langle u_{3},0.5,0.6\rangle \right\};$$

$$BP_{A_{4}}=\left\{\langle u_{1},0.4,0.6\rangle ,\langle u_{2},0.5,0.6\rangle ,\langle u_{3},0.6,0.7\rangle \right\};$$

$$BP_{A_{5}}=\left\{\langle u_{1},0.3,0.5\rangle ,\langle u_{2},0.5,0.6\rangle ,\langle u_{3},0.6,0.6\rangle \right\}$$

*and*

$$BP_{S}=\left\{\langle u_{1},0.4,0.8\rangle ,\langle u_{2},0.3,0.4\rangle ,\langle u_{3},0.6,0.7\rangle \right\}.$$

*The distance measure between BP-PiFSs using Equation (4) is calculated below:*

$$d\left(BP_{A_{1}},BP_{S}\right)=0.2000;\;d\left(BP_{A_{2}},BP_{S}\right)=0.1333;\;d\left(BP_{A_{3}},BP_{S}\right)=0.1667;$$

$$d\left(BP_{A_{4}},BP_{S}\right)=0.0667;\;d\left(BP_{A_{5}},BP_{S}\right)=0.1000.$$

*Similarly, the similarity measure between BP-PiFSs using Equation (6) is calculated below:*

$$S\left(BP_{A_{1}},BP_{S}\right)=0.4854;S\left(BP_{A_{2}},BP_{S}\right)=0.6131;S\left(BP_{A_{3}},BP_{S}\right)=0.6072;$$

$$S\left(BP_{A_{4}},BP_{S}\right)=6684;S\left(BP_{A_{5}},BP_{S}\right)=0.5485.$$

**Figure 2:**
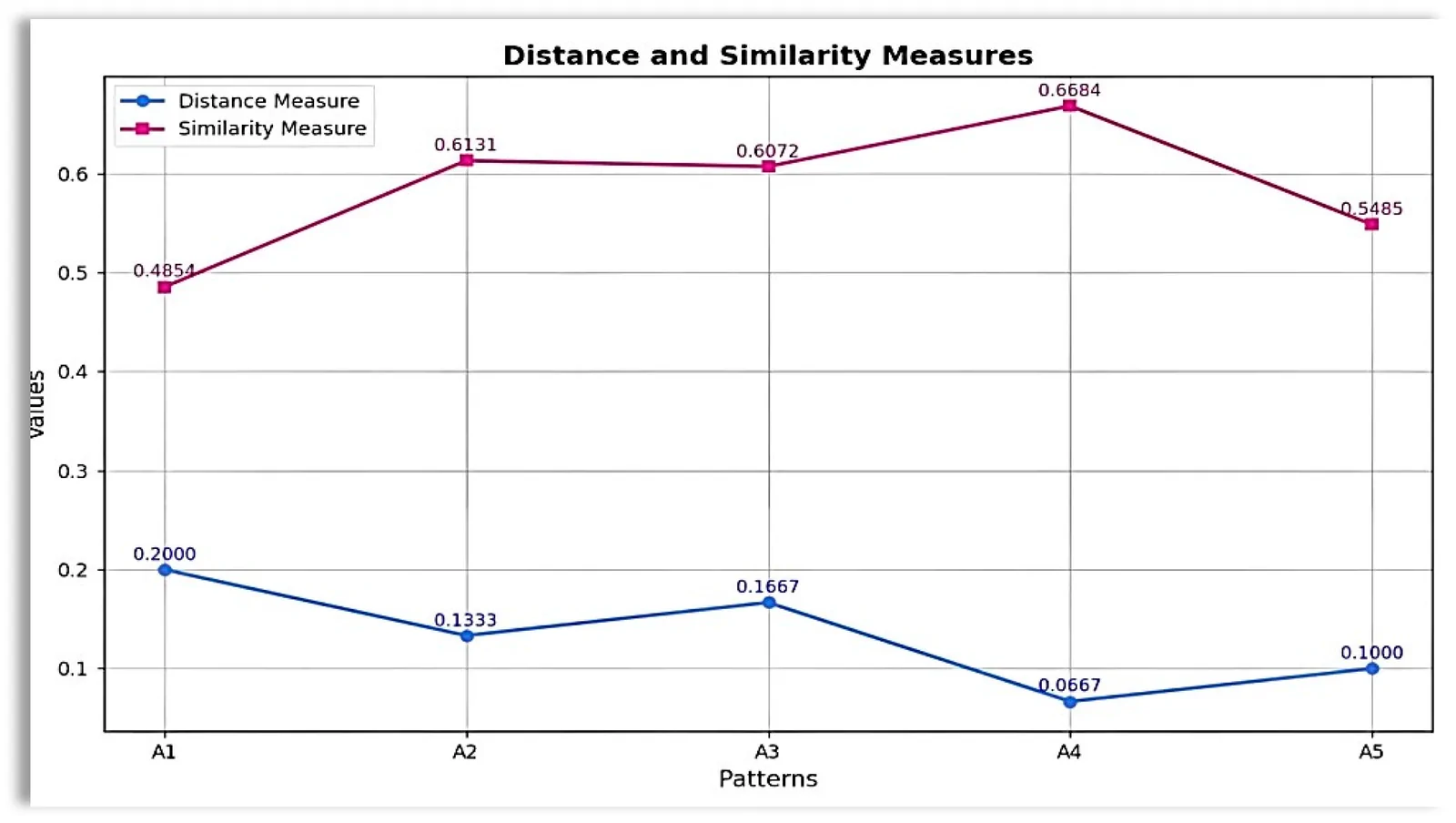
Comparison of distance and similarity measures between the patterns $BP_{A_{1}}$, $BP_{A_{2}}$, $BP_{A_{3}}$, $BP_{A_{4}}$, and $BP_{A_{5}}$ and sample $BP_{S}$.

These results are also displayed in Figure 2 below.

The above analysis shows the resemblance of BP-PiFS sample $BP_{S}$ with the pattern $BP_{A_{4}}$, on the basis of minimum distance and maximum similarity between two BP-PiFSs. From the framework of mechanical fault detection, this result means that $BP_{S}$ most likely represents a bearing defect, and this requires immediate attention to prevent further damage or failure of the machinery. This classification assists early fault detections, predictive maintenance, and reducing unexpected equipment downtime. In Example 1, we have shown that the proposed measures of Equations (4) and (8) are reliable and effective. Therefore, in the context of ET, the suggested measures are applicable and appropriate in the BP-PiFSs contexts.

### 5.2. A Comparison Analysis of the Existing and Suggested Distance Measures

For comparison analysis, we take the distance measures Equations (9) and (10) for PiFSs that were recently suggested by [45,46]. The existing measures given by [45,46] are as follows:


(9) $$d\left(A_{1},A_{2}\right)=\frac{1}{2n}\sum_{i=1}^{n}\left(\begin{array}{l} \left|\sqrt{\mu_{A_{1}}\left(u_{i}\right)}-\sqrt{\mu_{A_{2}}\left(u_{i}\right)}\right|+\\ \left|\sqrt{\nu_{A_{1}}\left(u_{i}\right)}-\sqrt{\nu_{A_{2}}\left(u_{i}\right)}\right|+\left|\sqrt{\eta_{A_{1}}\left(u_{i}\right)}-\sqrt{\eta_{A_{2}}\left(u_{i}\right)}\right| \end{array}\right)\;$$

(10) $$d\left(A_{1},A_{2}\right)=\sqrt{\frac{1}{2}\left(\begin{array}{l} \mu_{A_{1}}\left(u_{i}\right)\log\left(\frac{2\mu_{A_{1}}\left(u_{i}\right)}{\mu_{A_{1}}\left(u_{i}\right)+\mu_{A_{2}}\left(u_{i}\right)}\right)+\mu_{A_{2}}\left(u_{i}\right)\log\left(\frac{2\mu_{A_{2}}\left(u_{i}\right)}{\mu_{A_{1}}\left(u_{i}\right)+\mu_{A_{2}}\left(u_{i}\right)}\right)\\ +\nu_{A_{1}}\left(u_{i}\right)\log\left(\frac{2\nu_{A_{1}}\left(u_{i}\right)}{\nu_{A_{1}}\left(u_{i}\right)+\nu_{A_{2}}\left(u_{i}\right)}\right)+\nu_{A_{2}}\left(u_{i}\right)\log\left(\frac{2\nu_{A_{2}}\left(u_{i}\right)}{\nu_{A_{1}}\left(u_{i}\right)+\nu_{A_{2}}\left(u_{i}\right)}\right)\\ +\eta_{A_{1}}\left(u_{i}\right)\log\left(\frac{2\eta_{A_{1}}\left(u_{i}\right)}{\eta_{A_{1}}\left(u_{i}\right)+\eta_{A_{2}}\left(u_{i}\right)}\right)+\eta_{A_{2}}\left(u_{i}\right)\log\left(\frac{2\eta_{A_{2}}\left(u_{i}\right)}{\eta_{A_{1}}\left(u_{i}\right)+\eta_{A_{2}}\left(u_{i}\right)}\right) \end{array}\right)}$$

The PiFS patterns of Example 1 are taken for comparison analysis, which are:


$$A_{1}=\left\{\langle u_{1},0.2,0.1,0.3\rangle ,\langle u_{2},0.1,0.3,0.1\rangle ,\langle u_{3},0.4,0.3,0.2\rangle \right\};$$

$$A_{2}=\left\{\langle u_{1},0.4,0.1,0.3\rangle ,\langle u_{2},0.3,0.5,0.1\rangle ,\langle u_{3},0.2,0.4,0.2\rangle \right\};$$

$$A_{3}=\left\{\langle u_{1},0.3,0.2,0.1\rangle ,\langle z_{2},0.6,0.1,0.3\rangle ,\langle u_{3},0.5,0.1,0.3\rangle \right\};$$

$$A_{4}=\left\{\langle u_{1},0.4,0.3,0.1\rangle ,\langle u_{2},0.5,0.3,0.1\rangle ,\langle u_{3},0.6,0.1,0.2\rangle \right\};$$

$$A_{5}=\left\{\langle u_{1},0.3,0.1,0.4\rangle ,\langle u_{2},0.5,0.2,0.2\rangle ,\langle u_{3},0.6,0.1,0.3\rangle \right\}$$

and a sample S is represented as:

$$S=\left\{\langle u_{1},0.4,0.1,0.1\rangle ,\langle u_{2},0.3,0.4,0.2\rangle ,\langle u_{3},0.6,0.1,0.2\rangle \right\}$$

The purpose is to assign the unknown pattern S to an appropriate class by analyzing its closeness to the available samples. This analysis is shown in Table 1.

Table 1 shows the resemblance of S with the $A_{4}$ under the principle of minimum distance of two PiFS patterns. These results shows that both the proposed distance of Equation (4) and the existing distance proposed by [45,46] identifies the similar patterns, which attest the appropriateness of our proposed measure of Equation (4).

**Figure 3:**
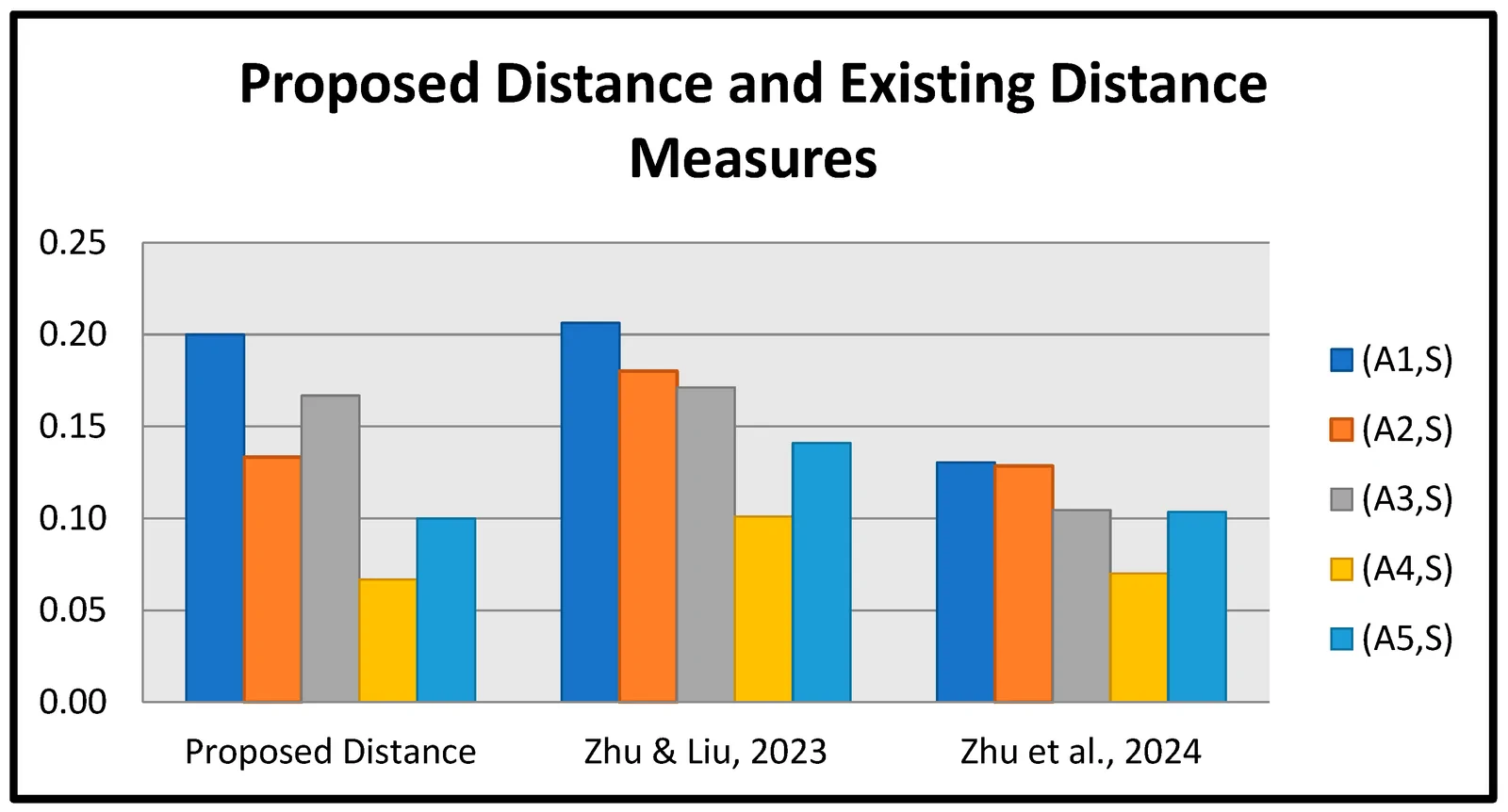
Comparison of proposed distance and existing distance measures [45,46] between the patterns $A_{1}$, $A_{2}$, $A_{3}$, $A_{4}$, $A_{5}$ and sample S.

These results are also displayed in Figure 3 below.

**Remark** **2.** *For the degenerated cases (1, 0, 0) and (0, 1, 0), Equation (10) proposed by [46] becomes undefined. Our proposed distance effectively addresses this issue, demonstrating the suitability of our approach compared to the existing method.*

In our everyday lives, we constantly encounter diverse forms of information that need to be organized and understood. Just as we group similar tasks or ideas to manage them more efficiently, clustering in data analysis serves a similar purpose. It involves recognizing patterns and grouped the data points based on their similarity, without prior knowledge of the group labels. This process is essential in uncovering hidden structures within large datasets and plays a vital role in simplifying complex information for better understanding and analysis. Therefore, we present a clustering analysis in the next section.

### 5.3. Application to Clustering

Clustering is a method used to separate data points into meaningful groups based on their similarity, so that members of the same cluster are closely related, while those in different clusters are less alike [34,35]. Hierarchical clustering is a machine learning method that organizes data into a tree-like structure. It works either by splitting large clusters into smaller ones (divisive) or by combining smaller clusters into larger ones (agglomerative). A common example is arranging books into genres and then grouping those genres into broader categories. This technique is widely used in fields such as medicine to group patients with similar symptoms, engineering to identify failure patterns, and biology to study gene relationships. It is also useful in materials science, image analysis, anomaly detection, and customer segmentation, helping researchers and businesses identify patterns and make better decisions.

Now, the proposed distance measure of Equation (4) and similarity measure of Equation (8) are used in cluster analysis. The main steps include:

Constructing a matrix by calculating the $d\left(BP_{A_{i}},BP_{A_{j}}\right)$ and $S\left(BP_{A_{i}},BP_{A_{j}}\right)$, using Equations (4) and (8).

The confidence level is $\;\tau \in \left[0,1\right]$.

Let $\chi_{ij}^{\tau }=\;\left\{\begin{array}{l} 0,\;\;\;d\left(BP_{A_{i}},BP_{A_{j}}\right)\;<\;\tau \\ 1,\;d\left(BP_{A_{i}},BP_{A_{j}}\right)\;\geq \;\tau \end{array}\right.$ and $\chi_{ij}^{\tau }=\;\left\{\begin{array}{l} 1,\;\;\;\;S\left(BP_{A_{i}},BP_{A_{j}}\right)\;<\;\tau \\ 0,\;S\left(BP_{A_{i}},BP_{A_{j}}\right)\;\geq \;\tau \end{array}\right.$, to construct the τ-cutting matrix $D_{\tau }={\left(\chi_{ij}^{\tau }\right)}_{n\times n}$.

(i) On the basis of $D_{\tau }={\left(\chi_{ij}^{\tau }\right)}_{n\times n}$, different clusters are obtained.

**Example** **2.** *To enhance the functionality, reliability, efficiency, and machinery fault diagnosis, engineers adopt a systematic approach that involves the continuous monitoring and evaluation of machinery performance. This approach is centered on the identification and classification of faults using seven critical diagnostic factors, each targeting a specific aspect of machine health. These factors cover both mechanical issues such as misalignment and overheating and electrical problems like voltage fluctuations or motor failure. By integrating these indicators, engineers can gain a holistic view of machine performance, allowing for timely detection of abnormalities before they escalate into critical failures. This diagnostic framework is particularly valuable for heavy-duty and industrial machinery, which operate under harsh conditions and are vital to uninterrupted production. Through early detection, the risk of costly downtime, major breakdowns, and safety hazards is significantly reduced. Moreover, this structured fault identification supports data-driven decision-making and lays the groundwork for predictive maintenance strategies. Ultimately, it ensures that machinery runs safely, efficiently, and economically over its operational lifespan. The seven key diagnostic factors are:*

(i) Vibration Analysis (A_1_): Detects abnormal vibration patterns that may indicate internal problems such as imbalance or bearing defects, often serving as the first sign of mechanical failure.
(ii) Temperature Monitoring (A_2_): Measures excessive heat generation in components, helping to identify friction, lubrication failure, or thermal stress that can lead to part deformation or seizure.
(iii) Noise Pattern Recognition (A_3_): Analyzes acoustic signals to pinpoint anomalies like grinding, knocking, or rattling, which are often signs of cracks or broken components.
(iv) Lubrication Condition (A_4_): Evaluates the quality, viscosity, and presence of lubricants to prevent metal-to-metal contact that accelerates wear and leads to catastrophic damage.
(v) Electrical Performance (A_5_): Monitors irregularities in current, voltage, and resistance, which can reveal issues like insulation breakdown, short circuits, or motor winding failures.
(vi) Operational History (A_6_): Considers past maintenance logs, usage frequency, and load cycles to determine how stress and age have contributed to current conditions.
(vii) Downtime Impact (A_7_): Assesses the financial and operational consequences of machine failure, including lost productivity, repair costs, and safety hazards.

To make sense of the vast data generated by these monitoring systems, clustering techniques are applied. These methods group similar machinery faults into identifiable patterns or clusters for example, one cluster may include faults caused by bearing wear associated with high vibration and heat, while another may involve electrical motor issues linked to irregular voltage signals. This classification enables engineers to identify underlying causes more efficiently and develop targeted maintenance strategies. The objective of clustering in this context is to simplify the system, reveal hidden patterns, and support predictive diagnostics. By transforming raw sensor and operational data into meaningful fault categories, clustering helps prioritize risks, streamline decision-making, and allocate maintenance resources more effectively.

The key seven diagnostic indicators are $A=\left\{A_{1},A_{2},\ldots ,A_{7}\right\}$ and expressed by PiFSs as:


$$A_{1}=\left\{\left(u_{1},\;0.2,0.1,\;0.3\right),\left(u_{2},0.1,\;0.3,0.1\right),\left(u_{3},0.4,\;0.3,0.2\right)\right\};$$

$$A_{2}=\left\{\left(u_{1},\;0.4,0.1,\;0.3\right),\left(u_{2},0.3,\;0.5,0.1\right),\left(u_{3},0.2,\;0.4,0.2\right)\right\};$$

$$A_{3}=\left\{\left(u_{1},\;0.3,\;0.2,0.1\right),\left(u_{2},0.6,\;0.1,0.3\right),\left(u_{3},0.5,\;0.1,0.3\right)\right\};$$

$$A_{4}=\left\{\left(u_{1},\;0.4,\;0.3,0.1\right),\left(u_{2},0.5,\;0.3,0.1\right),\left(z_{3},0.6,\;0.1,0.2\right)\right\};$$

$$A_{5}=\left\{\left(u_{1},\;0.3,\;0.1,0.4\right),\left(u_{2},0.5,\;0.2,0.2\right),\left(u_{3},0.6,\;0.1,0.3\right)\right\};$$

$$A_{6}=\left\{\left(u_{1},\;0.1,\;0.2,0.3\right),\left(u_{2},0.2,\;0.3,0.1\right),\left(u_{3},0.3,\;0.2,0.4\right)\right\};$$

$$A_{7}=\left\{\left(u_{1},\;0.4,\;0.1,0.1\right),\left(u_{2},0.3,\;0.4,0.2\right),\left(u_{3},0.6,0.1,\;0.2\right)\right\}.$$

The corresponding BP-PiFSs are:


$$BP_{A_{1}}=\left\{\left(u_{1},\;0.2,\;0.6\right),\left(u_{2},0.1,\;0.6\right),\left(u_{3},0.4,\;0.5\right)\right\};$$

$$BP_{A_{2}}=\left\{\left(u_{1},\;0.4,\;0.6\right),\left(u_{2},0.3,\;0.4\right),\left(u_{3},0.2,\;0.4\right)\right\};$$

$$BP_{A_{3}}=\left\{\left(u_{1},\;0.3,\;0.7\right),\left(u_{2},0.6,\;0.6\right),\left(u_{3},0.5,\;0.6\right)\right\};$$

$$BP_{A_{4}}=\left\{\left(u_{1},\;0.4,\;0.6\right),\left(u_{2},0.5,\;0.6\right),\left(u_{3},0.6,\;0.7\right)\right\};$$

$$BP_{A_{5}}=\left\{\left(u_{1},\;0.3,\;0.5\right),\left(u_{2},0.5,\;0.6\right),\left(u_{3},0.6,\;0.6\right)\right\};$$

$$BP_{A_{6}}=\left\{\left(u_{1},\;0.1,\;0.5\right),\left(u_{2},0.2,\;0.6\right),\left(u_{3},0.3,\;0.4\right)\right\};$$

$$BP_{A_{7}}=\left\{\left(u_{1},\;0.4,\;0.8\right),\left(u_{2},0.3,\;0.4\right),\left(u_{3},0.6,\;0.7\right)\right\}.$$

Using Equation (4), the constructed matrix is

$$\left[\begin{array}{ccccccc} 0 & & & & & & \\ 0.2000 & 0 & & & & & \\ 0.2333 & 0.2333 & 0 & & & & \\ 0.2667 & 0.2000 & 0.1000 & 0 & & & \\ 0.2333 & 0.2333 & 0.0667 & 0.0333 & 0 & & \\ 0.1000 & 0.1666 & 0.2666 & 0.3000 & 0.2667 & 0 & \\ 0.2000 & 0.1333 & 0.1667 & 0.0666 & 0.1000 & 0.2333 & 0 \end{array}\right]$$

The clustering results for the dataset $A=\left\{A_{1},A_{2},\ldots ,A_{7}\right\}$ with various values of τ levels are presented in Table 2.

Similarly, using Equation (8), the calculated similarity matrix is


$$\left[\begin{array}{ccccccc} 1 & & & & & & \\ 0.6093 & 1 & & & & & \\ 0.6386 & 0.4724 & 1 & & & & \\ 0.6837 & 0.5561 & 0.7621 & 1 & & & \\ 0.6456 & 0.4558 & 0.7402 & 0.8335 & 1 & & \\ 0.7658 & 0.6337 & 0.4899 & 0.5389 & 0.6573 & 1 & \\ 0.4854 & 0.6131 & 0.6072 & 0.6684 & 0.5485 & 0.3718 & 1 \end{array}\right]$$

The clustering results for the dataset $A=\left\{A_{1},A_{2},\ldots ,A_{7}\right\}$ with various values of τ levels are presented in Table 3.

The above example reveals the validity of Equations (4) and (8) in clustering. Based on the different values of τ, we use our suggested measures between two BP-PiFSs to generate hierarchical clusters for the data.

**Figure 4:**
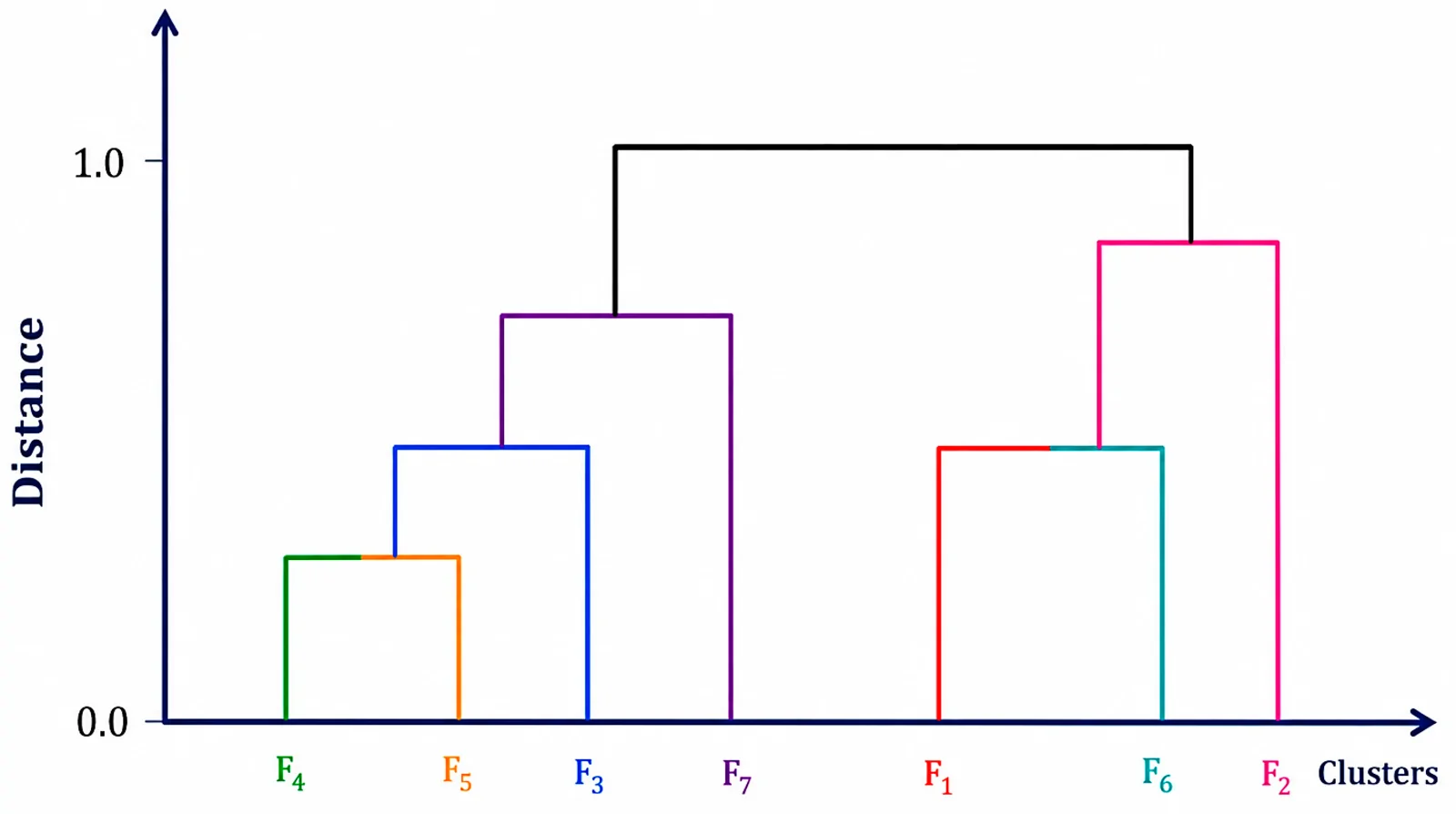
Clustering dendrogram obtaining from Equation (4).

Figure 4 illustrates the clustering dendrogram using Equation (4).

**Figure 5:**
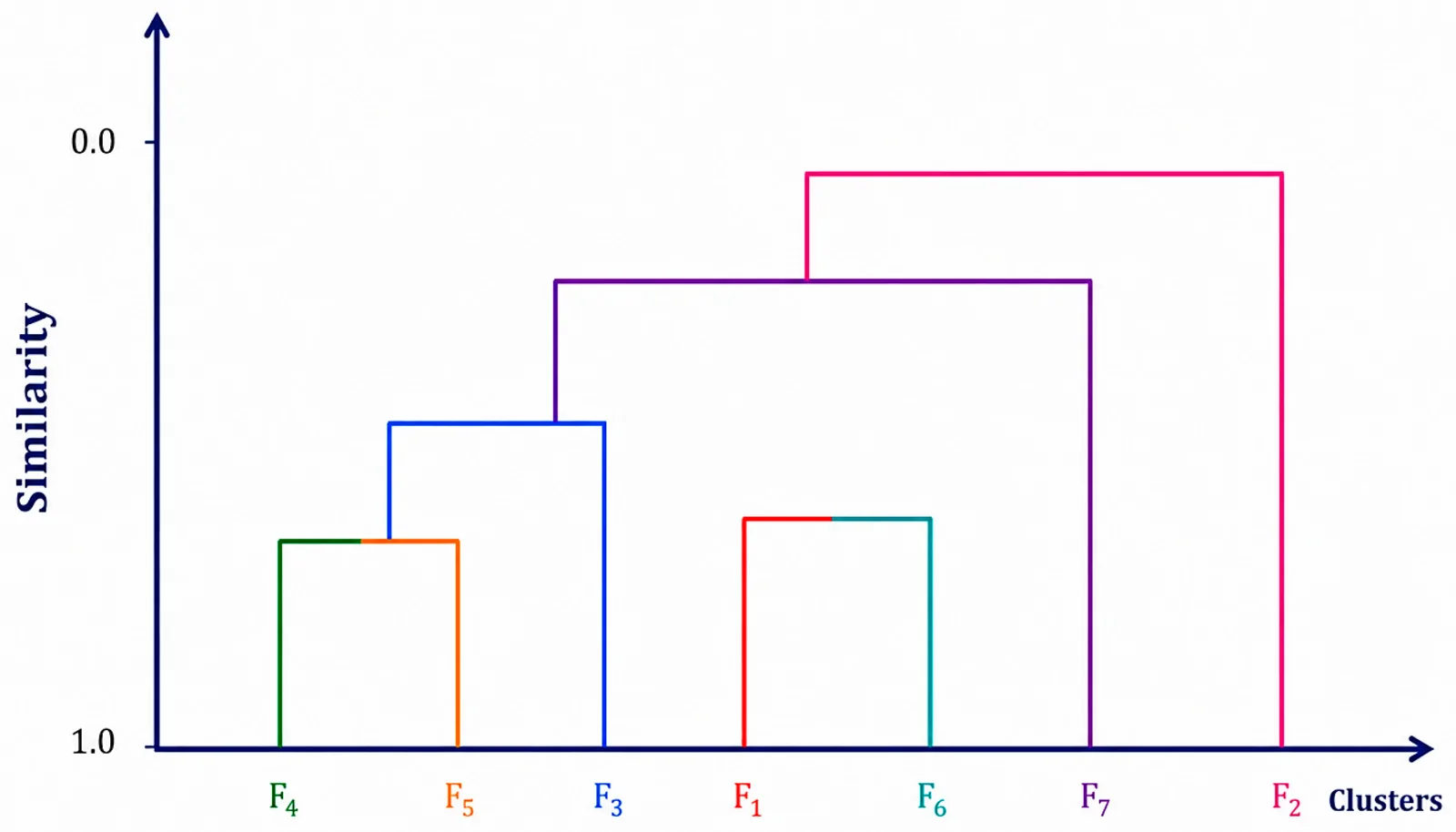
Clustering dendrogram obtaining from Equation (8).

Similarly, Figure 5 illustrates the clustering dendrogram using Equation (8).

Ultimately, this comprehensive clustering approach enhances not only fault detection and prevention but also boosts the reliability, safety, and cost-efficiency of heavy machinery operations across various engineering industries.

### 5.4. Application to Multi-Criteria Decision Making

Decision-making involves selecting the best option from available alternatives. Selection from a range of options is a common on daily basis. MCDM is a method used to compare options and select the best one based on available facts.

Complex problems can make decision-making difficult. Decision-making helps individuals choose the best option from several alternatives. To ensure logical and reliable choices, a structured model is needed. Such a model provides clear steps that support rational and informed decisions. In below section, classical SMART method is extended to establish the BP-SMART based on our proposed measure to tackle MCDM problems.

#### 5.4.1. New Belief and Plausible SMART Method

In this section, we use modified BP-SMART to identifying best alternative.

The SMART algorithm was initially introduced by [58,59] and stands as a practical and effective approach for MCDM. This approach depends on linear-valued functions for evaluating and ranking alternatives. Its versatility in handling both quantitative and qualitative criteria make it a compensatory technique widely applied across various contexts. Notably, it has been extensively employed in the assessment of vulnerability in critical facilities [60]. In 2017, there was a notable research study focused on the SMART for decision support [61].

Suppose $T=\left\{T_{1},T_{2},\ldots ,T_{p}\right\}$ be some set of alternatives where $T_{m}$ represents the mth alternative $\left(m=1,2,\ldots ,p\right)$. Similarly, let a set of criteria for alternatives, $K=\left\{K_{1},K_{2},\ldots ,K_{q}\right\}$, where $K_{n}$ represents the nth criterion $\left(n=1,2,\ldots ,q\right).$ Consequently, the BP-SMART method’s implementation steps are further elaborated as follows:

Step-1: Creation of Belief and Plausible Decision Matrix: Consider a set of alternatives denoted as $T=\left\{T_{1},T_{2},\ldots ,T_{p}\right\}$ on criteria, $K=\left\{K_{1},K_{2},\ldots ,K_{q}\right\}$. Suppose the decision matrix represented as $\hat{D}={\left[s_{mn}\right]}_{p\times q}=\left[B\left(u\right),P\left(u\right)\right]$, where $B\left(u\right)$, and $P\left(u\right)$ denotes degree of belief and degree of plausibility. The decision matrix can be formulated as:


$$\begin{array}{l} \;\;\;\;\;\;\;\;\;\;\;\;\;\;\;\;\;\;\;\;\;\;\;\;\;\;\;\;\;\;\;\;\;\;\;\;\;\begin{array}{cccc} K_{1} & K_{2} & \ldots & K_{q} \end{array}\\ \hat{D}={\left[s_{mn}\right]}_{p\times q}=\;\begin{array}{c} T_{1}\\ T_{2}\\ \vdots \\ T_{p} \end{array}\left[\begin{array}{cccc} s_{11} & s_{12} & \ldots & s_{1q}\\ s_{21} & s_{22} & \ldots & s_{2q}\\ \ldots & \ldots & \ddots & \vdots \\ s_{p1} & s_{p2} & \ldots & s_{pq} \end{array}\right] \end{array}$$

Step-2: Criteria Weight: We determine the weight of each criterion using different methods. Let us assume that the weights assigned to the criteria are $w_{j}$ can be obtained using the formula:

(11) $$w_{n}=\frac{\left[max\left(B_{mn}\left(u\right)\right)+min\left(P_{mn}\left(u\right)\right)/2\right]}{\sum_{m=1}^{n}\left[max\left(B_{mn}\left(u\right)\right)+min\left(P_{mn}\left(u\right)\right)/2\right]},\;n=1,2,\ldots ,q$$

with, $\sum_{n=1}^{q}w_{n}=1$ for $0\leq w_{n}\leq 1$.

Step-3: Normalized Matrix: In this stage, we develop the normalized matrix $N_{mn}={\left[a_{mn}\right]}_{p\times q}$ as:

$$N_{mn}=\begin{array}{c} T_{1}\\ T_{2}\\ \vdots \\ T_{p} \end{array}\left[\begin{array}{cccc} a_{11} & a_{12} & \ldots & a_{1q}\\ a_{21} & a_{22} & \ldots & a_{2q}\\ \ldots & \ldots & \ddots & \vdots \\ a_{p1} & a_{p2} & \ldots & a_{pq} \end{array}\right],$$

where $\left(m=1,2,\ldots ,p\right)$, and $\left(n=1,2,\ldots ,q\right).$

Here,

(12) $$a_{pq}=\frac{d\left[max\left(K_{q}\right),s_{p}\;Value\right]}{d\left[max\left(K_{q}\right),min\left(K_{q}\right)\right]}$$

for proposed distance measure and

(13) $$a_{pq}=\frac{S\left[max\left(K_{q}\right),s_{p}\;Value\right]}{S\left[max\left(K_{q}\right),min\left(K_{q}\right)\right]}$$

for proposed similarity measure, respectively.

Step-4: Develop the Normalized weighted-matrix: We then calculate the normalized weighted-matrix as:

$${\overset{w}{N}}_{mn}={\left[w_{m}K_{n}\right]}_{p\times q}$$

where $\left(n=1,2,\ldots ,q\right)$.

$${\overset{w}{N}}_{mn}=\left[\begin{array}{cccc} w_{1}K_{1} & w_{2}K_{2} & \ldots & w_{m}K_{n} \end{array}\right]\;\;,$$

where $\left(m=1,2,\ldots ,p\right)$ and $\left(n=1,2,\ldots ,q\right)$.

Step-5: Ranking of Alternatives: The weighted-matrix is employed to choose the optimal choice based on predefined criteria by adding the row values of normalized weighted-matrix as:

$$T_{m}=\sum_{n=1}^{q}w_{m}K_{n},$$

where $\left(m=1,2,\ldots ,p\right)$.

**Figure 6:**
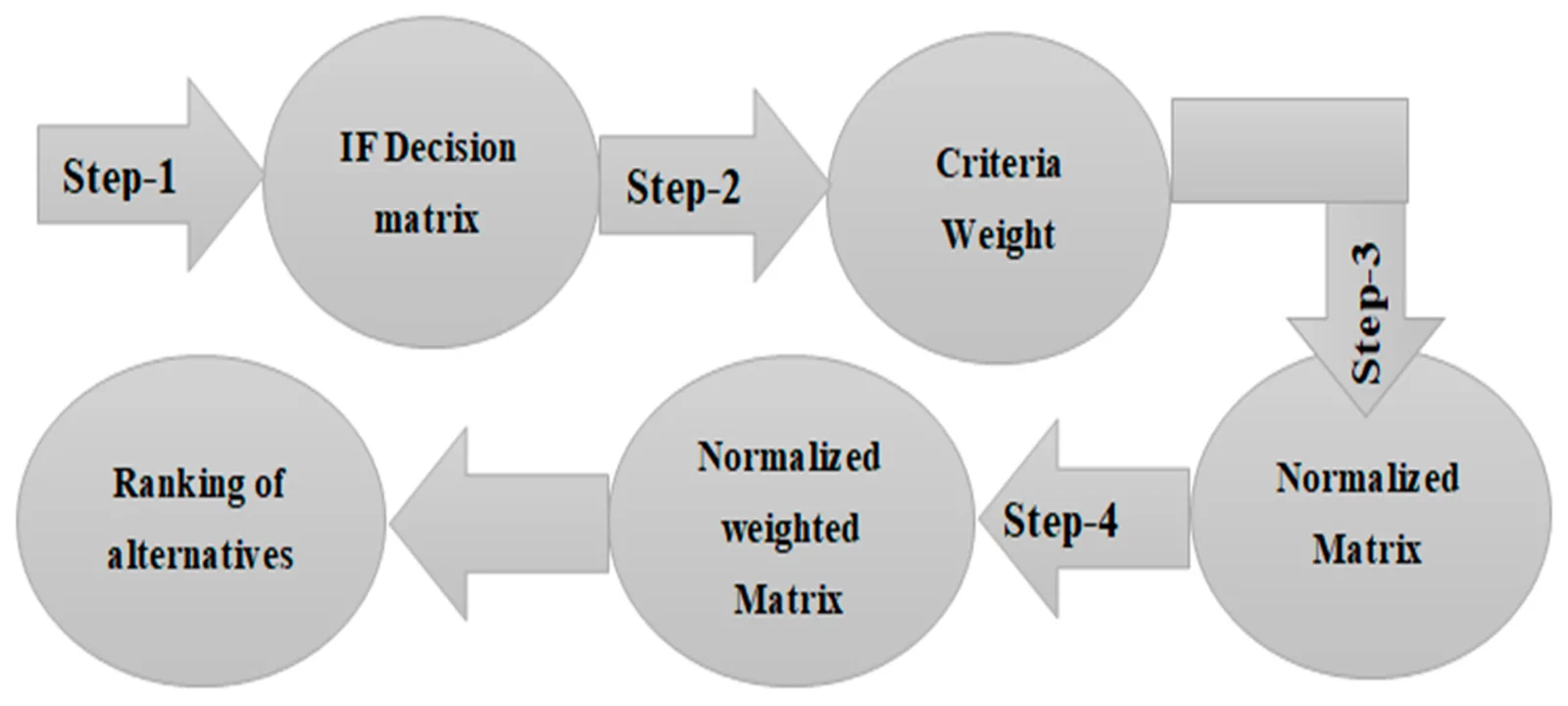
Flowchart for the BP-SMART algorithm.

The stepwise flowchart of BP-SMART algorithm is demonstrated in Figure 6 as:

The algorithm is applied to demonstrate its effectiveness in real-world daily problems.

**Example** **3.** *Antenna design and analysis are fundamental to the success of satellite communication systems, as they directly influence signal reliability, data transmission quality, and overall performance of the system. Beyond basic functionality, modern antenna designs aim to address complex challenges such as optimizing configurations, improving performance metrics, and managing uncertainties arising from environmental and operational conditions. Satellite communication systems, in particular, rely heavily on antennas that can maintain robust link reliability and ensure efficient data flow across vast distances. This requires antennas capable of precise beam shaping and pointing accuracy to overcome obstacles like atmospheric turbulence, positional shifts, and interference.*

To achieve these goals, a systematic approach to antenna design and evaluation is necessary. Advanced techniques such as BP-SMART technique are used to rank and select the most suitable antenna configurations based on multiple criteria. Additionally, Picture fuzzy controllers play a pivotal role in managing uncertainties, providing a sophisticated modeling framework that accounts for variations in atmospheric and positional conditions. These controllers enable precise adjustments in beam shaping and pointing, ensuring the antenna adapts to real-time challenges and maintains optimal performance.

##### Role of Picture Fuzzy Controllers in Managing Uncertainty

Picture fuzzy logic enhances decision-making process by effectively managing uncertainties in expert evaluations and real-world conditions. Unlike traditional fuzzy systems, Picture fuzzy controllers offer a refined approach by including degree of membership, non-membership, neutrality and hesitation which enables precise modeling of complex, uncertain scenarios. This is especially valuable for satellite communication, where antennas must adapt to varying atmospheric conditions such as rain fade, temperature fluctuations, and interference from solar activity.

These controllers optimize beam shaping and pointing accuracy, ensuring the antenna maintains its alignment and communication link even in challenging environments. By integrating Picture fuzzy logic with BP-SMART, engineers can systematically rank the antenna configurations to select the most robust and efficient design for satellite communication systems. This comprehensive approach not only improves link reliability and antenna performance but also ensures resilience against uncertainties, enabling high-quality, uninterrupted communication in a variety of applications.

#### 5.4.2. An MCDM Approach for Optimizing Antenna Configurations

BP-SMART is a powerful MCDM method that systematically evaluates antenna designs by analyzing them against predefined criteria. This approach is particularly useful for satellite communication, where antennas must balance multiple performance factors such as gain, beam-width, efficiency, and cost. For example, in designing satellite communication systems, engineers often assess various antenna types as alternatives, which are:

T_1_: Parabolic Reflector Antennas: These types of antennas are known for their high gain and precise directional focus, and these are ideal for long-distance satellite communication.
T_2_: Phased Array Antennas: They are highly adaptable with advanced beam-forming capabilities, and these antennas excel in dynamic environments.
T_3_: Helical Antennas: They are specially known for their simplicity and consistent performance, especially under fluctuating conditions.
T_4_: Micro-strip Patch Antennas: They are compact and cost-effective, and they are suitable for applications with space and weight constraints.

These antenna types are evaluated under some key criteria such as:

K_1_: Cost: This criterion reflects the financial feasibility of the design.
K_2_: Gain and Beam-width: This measures the signal strength and coverage capability of antennas and assesses directionality and the ability to cover desired areas.
K_3_: Resilience to Uncertainties: Evaluating performance stability under environmental challenges like atmospheric disruptions or positional inaccuracies.

The choice of antenna design is one of the most crucial factors for satellite communication systems. The evaluation of each alternative $T_{i}$ is carried out using expert judgments expressed in the form of PiFSs as:

$$T_{1}=\left\{\langle K_{1},\left(0.4,0.3,0.2\right)\rangle ,\langle K_{2},\left(0.6,0.1,0.1\right)\rangle ,\langle K_{3},\left(0.5,0.2,0.2\right)\rangle \right\}\;;$$

$$T_{2}=\left\{\langle K_{1},\left(0.6,0.1,0.0\right)\rangle ,\langle K_{2},\left(0.5,0.1,0.4\right)\rangle ,\langle K_{3},\left(0.3,0.1,0.1\right)\rangle \right\}\;;$$

$$T_{3}=\left\{\langle K_{1},\left(0.3,0.3,0.2\right)\rangle ,\langle K_{2},\left(0.4,0.5,0.1\right)\rangle ,\langle K_{3},\left(0.5,0.3,0.2\right)\rangle \right\}\;;$$

$$T_{4}=\left\{\langle K_{1},\left(0.7,0.1,0.1\right)\rangle ,\langle K_{2},\left(0.3,0.2,0.1\right)\rangle ,\langle K_{3},\left(0.6,0.1,0.2\right)\rangle \right\}\;.$$

Each alternative is expressed in the form of BP-PiFSs as:


$$BP_{T_{1}}=\left\{\langle K_{1},\left(0.4,0.5\right)\rangle ,\langle K_{2},\left(0.6,0.8\right)\rangle ,\langle K_{3},\left(0.5,0.6\right)\rangle \right\}\;;$$

$$BP_{T_{2}}=\left\{\langle K_{1},\left(0.6,0.9\right)\rangle ,\langle K_{2},\left(0.5,0.5\right)\rangle ,\langle K_{3},\left(0.3,0.8\right)\rangle \right\}\;;$$

$$BP_{T_{3}}=\left\{\langle K_{1},\left(0.3,0.5\right)\rangle ,\langle K_{2},\left(0.4,0.4\right)\rangle ,\langle K_{3},\left(0.5,0.5\right)\rangle \right\}\;;$$

$$BP_{T_{4}}=\left\{\langle K_{1},\left(0.7,0.8\right)\rangle ,\langle K_{2},\left(0.3,0.7\right)\rangle ,\langle K_{3},\left(0.6,0.7\right)\rangle \right\}\;.$$

Step-1 The Belief and Plausible Picture fuzzy decision matrix is

$$\begin{array}{l} \;\;\;\;\;\;\;\;\;\;\;\;\;\begin{array}{ccc} K_{1} & \;\;\;\;\;\;\;\;K_{2}\;\;\;\;\;\; & K_{3} \end{array}\\ \hat{D}=\begin{array}{c} T_{1}\\ T_{2}\\ T_{3}\\ T_{4} \end{array}\left[\begin{array}{ccc} \langle 0.4,0.5\rangle & \langle 0.6,0.8\rangle & \langle 0.5,0.6\rangle \\ \langle 0.6,0.9\rangle & \langle 0.5,0.5\rangle & \langle 0.3,0.8\rangle \\ \langle 0.3,0.5\rangle & \langle 0.4,0.4\rangle & \langle 0.5,0.5\rangle \\ \langle 0.7,0.8\rangle & \langle 0.3,0.7\rangle & \langle 0.6,0.7\rangle \end{array}\right] \end{array}$$

Step-2: The criteria weights of each criterion are calculated using Equation (11) as:

$$w_{1}=0.364,\;\;\;\;\;\;w_{2}=0.303,\;\;\;\;\;\;w_{3}=0.333.$$

(i) Using proposed distance measure

Step 3: Normalized Matrix

Normalized matrix can be developed using Equation (12) as:

$$\begin{array}{l} \;\;\;\;\;\;\;\;\;\;\;\;\;\;\begin{array}{ccc} \;\;K_{1} & \;\;\;\;\;K_{2} & \;\;\;\;\;\;\;K_{3} \end{array}\\ N=\begin{array}{c} T_{1}\\ T_{2}\\ T_{3}\\ T_{4} \end{array}\left[\begin{array}{ccc} 0.7500 & 0 & 0.3333\\ 0.2500 & 0.3333 & 1\\ 1 & 0.6667 & 0.3333\\ 0 & 1 & 0 \end{array}\right] \end{array}$$

Step 4: Normalized Weighted-Matrix

$$\overset{w}{N}=\left[w_{m}K_{n}\right]=\begin{array}{c} T_{1}\\ T_{2}\\ T_{3}\\ T_{4} \end{array}\left[\begin{array}{cccc} \;K_{1} & K_{2} & \;K_{3} & \mathrm{Sum}\\ 0.2727 & 0 & 0.1109 & 0.3836\\ 0.0909 & 0.1011 & 0.3333 & 0.5253\\ 0.3636 & 0.2021 & 0.1109 & 0.6768\\ 0 & 0.3030 & 0 & 0.0303 \end{array}\right]$$

Step 5 Ranking: For distance, we choose the minimum value, so the final ranking is:

$$T_{4}≻T_{1}≻T_{2}≻T_{3}$$

(ii) Using Proposed Similarity Measure

Step 3: Normalized matrix can be established using Equation (13) as:

$$\begin{array}{l} \;\;\;\;\;\;\;\;\;\;\;\;\;\;\begin{array}{ccc} \;\;K_{1} & \;\;\;\;\;K_{2} & \;\;\;\;\;\;\;\;K_{3} \end{array}\\ N=\begin{array}{c} T_{1}\\ T_{2}\\ T_{3}\\ T_{4} \end{array}\left[\begin{array}{ccc} 1.1680 & 1.5580 & 1.1230\\ 2.4681 & 0.7230 & 1\\ 1 & 0.4620 & 0.8960\\ 3.1830 & 1 & 1.5581 \end{array}\right] \end{array}$$

Step 4: The normalized weighted-matrix will be

$$\overset{w}{N}=\left[w_{m}K_{n}\right]=\begin{array}{c} T_{1}\\ T_{2}\\ T_{3}\\ T_{4} \end{array}\left[\begin{array}{cccc} \;K_{1} & K_{2} & \;K_{3} & \mathrm{Sum}\\ 0.4247 & 0.4720 & 0.3743 & 1.2710\\ 0.8974 & 0.2191 & 0.3333 & 1.4498\\ 0.3636 & 0.1399 & 0.2986 & 0.8021\\ 1.1573 & 0.3030 & 0.5193 & 1.9796 \end{array}\right]$$

Step 5: For similarity, we choose the maximum value, so the final ranking is:

$$T_{4}≻T_{2}≻T_{1}≻T_{3}$$

This ranking is displayed in below Table 4.

**Figure 7:**
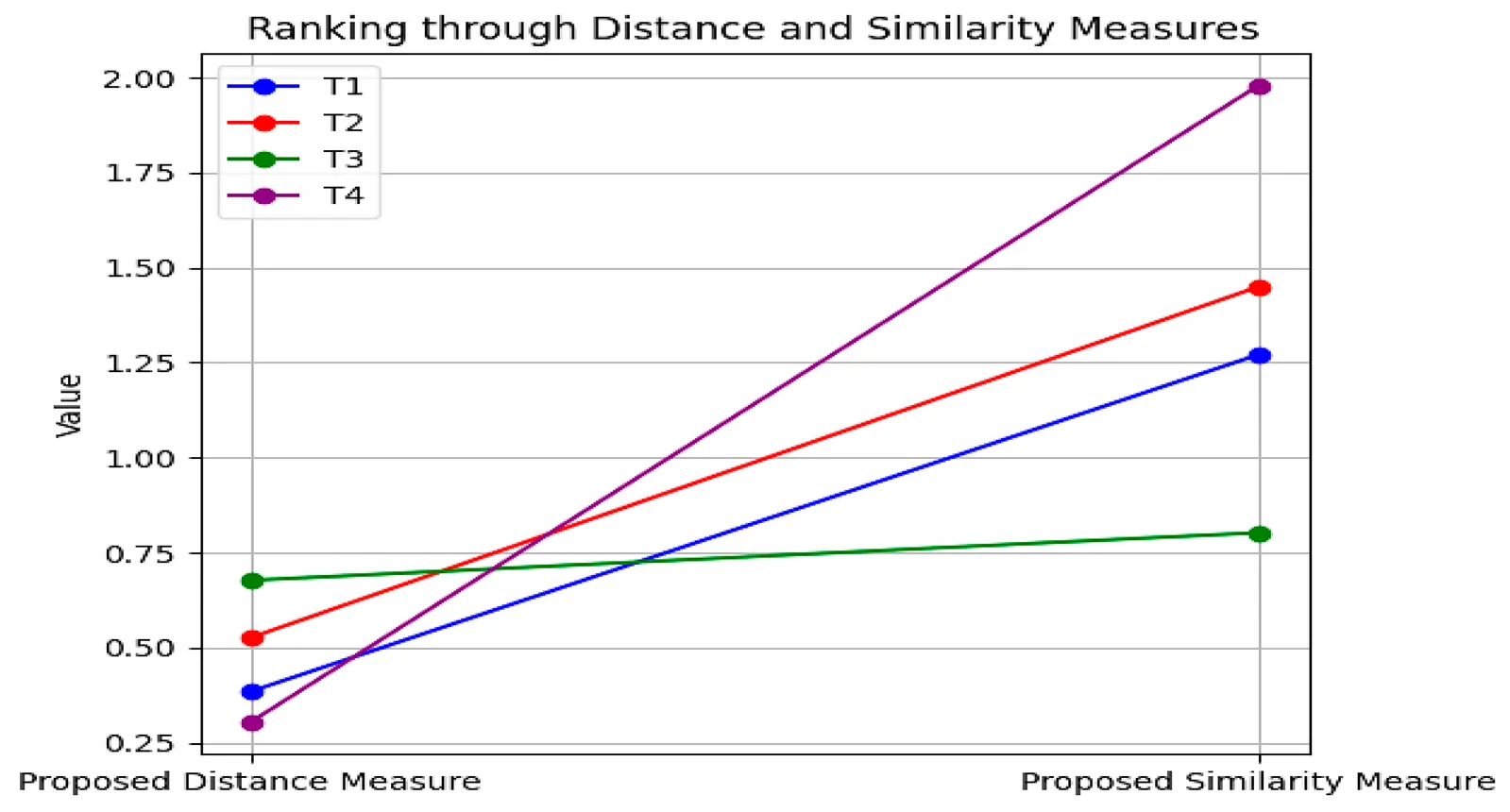
Rankings based on proposed distance and similarity measures.

Table 4 clearly depicts that $T_{4}$ is the best antenna design under given set of criteria. This ranking is shown in Figure 7 as:

Figure 7 demonstrates the final ranking of alternatives using our proposed measures of Equation (4) and Equation (6), respectively, and final ranking clearly shows no conflicts except $T_{2}$ and $T_{1}$. This might be happened due some round off values or noisy data. However, our objective was to identify the best antenna design under the given criteria. Both methods are agreed to select alternative $T_{4}$ as the best antenna design under the set criteria.

#### 5.4.3. Comparison of BP-SMART Algorithm with TOPSIS and GRA

Our Belief and Plausible Picture fuzzy decision matrix is

$$\begin{array}{l} \;\;\;\;\;\;\;\;\;\;\;\;\;\;\begin{array}{ccc} K_{1} & \;\;\;\;\;\;K_{2}\;\;\;\;\;\; & K_{3} \end{array}\\ \hat{D}=\begin{array}{c} T_{1}\\ T_{2}\\ T_{3}\\ T_{4} \end{array}\left[\begin{array}{ccc} \langle 0.4,0.5\rangle & \langle 0.6,0.8\rangle & \langle 0.5,0.6\rangle \\ \langle 0.6,0.9\rangle & \langle 0.5,0.5\rangle & \langle 0.3,0.8\rangle \\ \langle 0.3,0.5\rangle & \langle 0.4,0.4\rangle & \langle 0.5,0.5\rangle \\ \langle 0.7,0.8\rangle & \langle 0.3,0.7\rangle & \langle 0.6,0.7\rangle \end{array}\right] \end{array}$$

**Figure 8:**
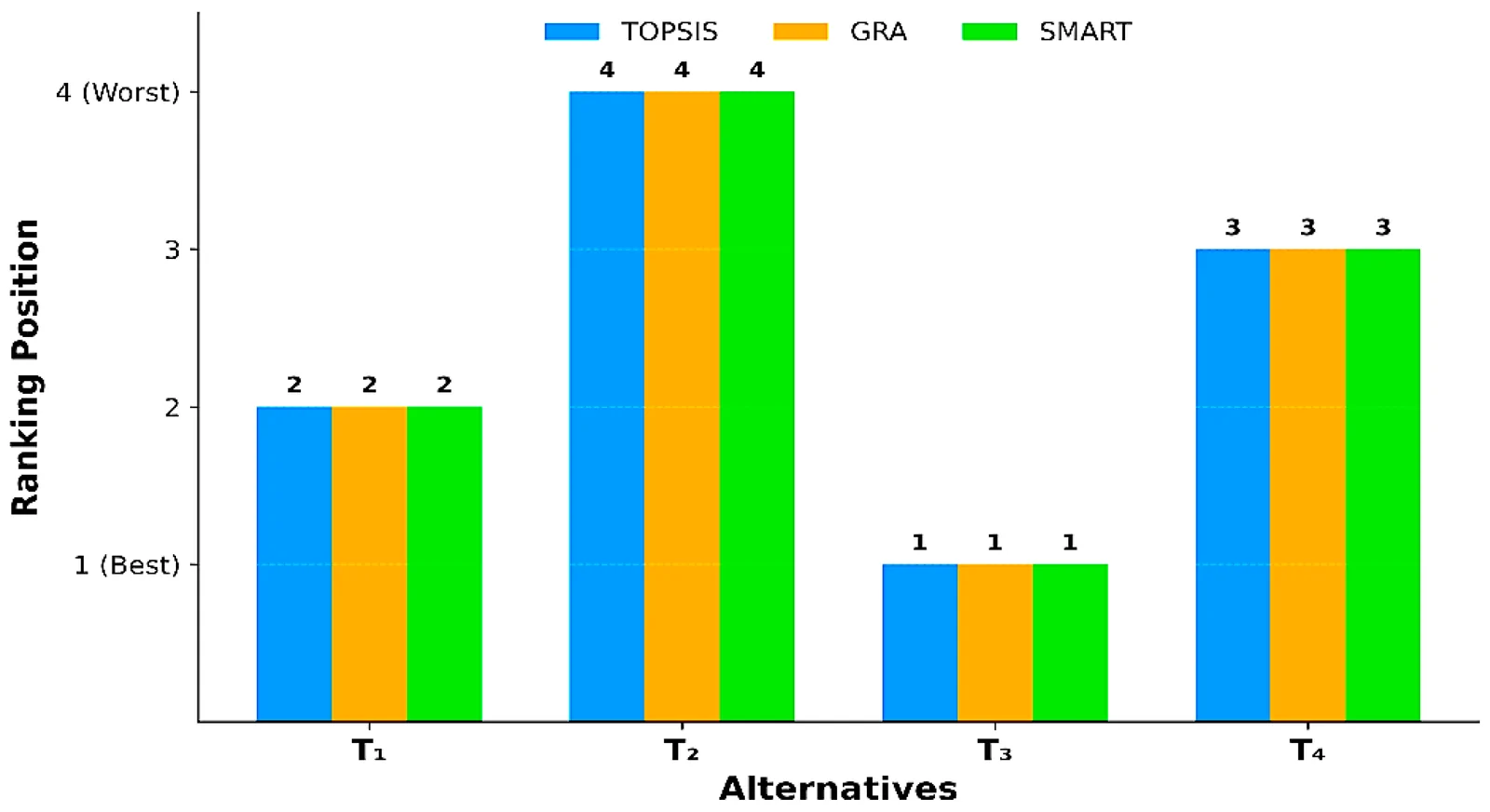
Comparison analysis of BP-SMART with TOPSIS and GRA algorithm.

By keeping the same weights as $w_{1}=0.364$, $w_{2}=0.303$, and $w_{3}=0.333$, our final results are shown in Table 5 and Figure 8.

A comparative analysis of the BP-SMART, TOPSIS, and GRA methods is shown in Table 5, which reveals complete consistency in the obtained rankings. All three approaches produce the same ranking results, with $T_{4}$ identified as the best alternative. This result demonstrates the robustness and reliability of the proposed decision-making framework across different MCDM techniques.

#### 5.4.4. Sensitivity Analysis

In the proposed BP-SMART model, the criterion weights are not assigned subjectively or manually. Instead, they are objectively determined using Equation (11), which adjusts the weights according to the characteristics of the evaluation data. Therefore, the weighting process is systematic and data-driven rather than based on expert preference. To clarify this point, the revised manuscript explicitly explains that Equation (11) is employed to compute the criterion weights, ensuring an objective and consistent weighting scheme for the decision-making process.

#### 5.4.5. Limitations of the Proposed Study

The present study primarily emphasizes the theoretical development and validation of the proposed BP-PiFSs framework through representative numerical examples and practical applications. Although the framework is applicable to complex real-world decision-making problems, its validation on large-scale, publicly available benchmark datasets has not been investigated in this work. Consequently, the scalability and generalizability of the proposed framework across diverse large-scale applications remain to be examined in future studies.

## 6. Conclusions and Future Work

The present work strengthens the theoretical foundation and practical applicability of PiFSs through the development of reliable distance and similarity measures for BP-PiFSs. These measures address existing methodological limitations and enhance their practical applicability, as demonstrated through extensive numerical analyses. The proposed method is applied in clustering problems. PiFSs play an important role in antenna applications due to their capability to represent uncertainty, vagueness, and multiple conflicting objectives encountered in real-world scenarios. To evaluate the practical performance of the proposed methods, the traditional SMART method is extended to the BP-SMART model. Additionally, the BP-SMART method is applied to antenna design and analysis to enhance performance, optimize configurations, and handle uncertainty in various applications, such as satellite communication to improve link reliability and optimize antenna performance for satellite communication systems. Picture fuzzy controllers help manage beam shaping and pointing accuracy under varying atmospheric and positional uncertainties. The proposed framework enhances the management of complex uncertain scenarios, facilitating more accurate decision-making, diagnostics, and pattern recognition in diverse applications. Future research will focus on validating the proposed framework using large-scale, publicly available benchmark datasets to further demonstrate its scalability and generalizability.

## Figures and Tables

**Table 1:** Comparison analysis of proposed and existing distances.

Proposed Distance d(BP_1_, BP_5_)	Existing Distance [45]	Existing Distance of [46]
d(BP_1_, BP_5_) = 0.2000	d(A_1_, S) = 0.2063	d(A_1_, S) = 0.1304
d(BP_2_, BP_5_) = 0.1333	d(A_2_, S) = 0.1801	d(A_2_, S) = 0.1285
d(BP_3_, BP_5_) = 0.1667	d(A_3_, S) = 0.1712	d(A_3_, S) = 0.1046
d(BP_4_, BP_5_) = 0.0667	d(A_4_, S) = 0.1011	d(A_4_, S) = 0.0701
d(BP_5_, BP_6_) = 0.1000	d(A_5_, S) = 0.1410	d(A_5_, S) = 0.1034

**Table 2:** Distance-based cluster analysis for different values of τ.

Confidence Levels	Clusters
0.0000 < τ ≤ 0.0333	{A_1_}, {A_2_}, {A_3_}, {A_4_}, {A_5_}, {A_6_}, {A_7_}
0.0333 < τ ≤ 0.0666	{A_1_}, {A_2_}, {A_3_}, {A_4_, A_5_}, {A_6_}, {A_7_}
0.0666 < τ ≤ 0.1000	{A_1_}, {A_2_}, {A_3_, A_4_, A_5_}, {A_6_}, {A_7_}
0.1000 < τ ≤ 0.1333	{A_1_, A_6_}, {A_2_}, {A_3_, A_4_, A_5_}, {A_7_}
0.1333 < τ ≤ 0.1667	{A_1_, A_6_, A_7_}, {A_2_}, {A_3_, A_4_, A_5_}
0.1667 < τ ≤ 0.2000	{A_1_, A_2_, A_6_}, {A_3_, A_4_, A_5_, A_7_}
0.2000 < τ ≤ 1.0000	{A_1_, A_2_, A_3_, A_4_, A_5_, A_6_, A_7_}

**Table 3:** Similarity-based clustering for different values of τ.

Confidence Levels	Clusters
1.0000 < τ ≤ 0.8333	{A_1_}, {A_2_}, {A_3_}, {A_4_}, {A_5_}, {A_6_}, {A_7_}
0.8333 < τ ≤ 0.7658	{A_1_}, {A_2_}, {A_3_}, {A_4_}, {A_5_}, {A_6_, A_7_}
0.7658 < τ ≤ 0.7621	{A_1_, A_6_}, {A_2_}, {A_3_}, {A_4_, A_5_}, {A_7_}
0.7621 < τ ≤ 0.7402	{A_1_, A_6_}, {A_2_}, {A_3_, A_4_, A_5_}, {A_7_}
0.6837 < τ ≤ 0.6684	{A_1_, A_3_, A_4_, A_5_, A_6_}, {A_2_}, {A_7_}
0.6684 < τ ≤ 0.6573	{A_1_, A_2_, A_3_, A_4_, A_6_}, {A_5_}, {A_7_}
0.6337 < τ ≤ 0.0000	{A_1_, A_2_, A_3_, A_4_, A_5_, A_6_, A_7_}

**Table 4:** Ranking of alternatives.

	Ranking	Best Alternative
$d\left(T_{1},T_{2}\right)$	T_4_ ≻ T_1_ ≻ T_2_ ≻ T_3_	T_4_
$S\left(T_{1},T_{2}\right)$	T_4_ ≻ T_1_ ≻ T_2_ ≻ T_3_	T_4_

**Table 5:** Comparison analysis with other methods.

Method	Ranking	Best Alternative
BP-SMART	T_4_ ≻ T_1_ ≻ T_2_ ≻ T_3_	T_4_
TOPSIS	T_4_ ≻ T_1_ ≻ T_2_ ≻ T_3_	T_4_
GRA	T_4_ ≻ T_1_ ≻ T_2_ ≻ T_3_	T_4_

## Data Availability

All relevant data are included within the article.
